# Whole genome sequences reveal the *Xanthomonas perforans* population is shaped by the tomato production system

**DOI:** 10.1038/s41396-021-01104-8

**Published:** 2021-09-06

**Authors:** Jeannie M. Klein-Gordon, Sujan Timilsina, Yanru Xing, Peter Abrahamian, Karen A. Garrett, Jeffrey B. Jones, Gary E. Vallad, Erica M. Goss

**Affiliations:** 1 Department of Plant Pathology, IFAS, University of Florida, Gainesville, FL USA; 2 Emerging Pathogens Institute, University of Florida, Gainesville, FL USA; 3 Food Systems Institute, University of Florida, Gainesville, FL USA; 4 Gulf Coast Research and Education Center, IFAS, University of Florida, Balm, FL USA; 5 USDA-ARS, Beltsville Agricultural Research Center, Molecular Plant Pathology Laboratory, Beltsville, MD USA

**Keywords:** Applied microbiology, Population genetics, Microbial ecology, Microbial ecology

## Abstract

Modern agricultural practices increase the potential for plant pathogen spread, while the advent of affordable whole genome sequencing enables in-depth studies of pathogen movement. Population genomic studies may decipher pathogen movement and population structure as a result of complex agricultural production systems. We used whole genome sequences of 281 *Xanthomonas perforans* strains collected within one tomato production season across Florida and southern Georgia fields to test for population genetic structure associated with tomato production system variables. We identified six clusters of *X. perforans* from core gene SNPs that corresponded with phylogenetic lineages. Using whole genome SNPs, we found genetic structure among farms, transplant facilities, cultivars, seed producers, grower operations, regions, and counties. Overall, grower operations that produced their own transplants were associated with genetically distinct and less diverse populations of strains compared to grower operations that received transplants from multiple sources. The degree of genetic differentiation among components of Florida’s tomato production system varied between clusters, suggesting differential dispersal of the strains, such as through seed or contaminated transplants versus local movement within farms. Overall, we showed that the genetic variation of a bacterial plant pathogen is shaped by the structure of the plant production system.

## Introduction

Human activities have accelerated long-distance movement of microbes, changing global distributions of microbes as well as genetic variation within and among local populations. Modern agricultural practices rely on the movement of plant materials which can facilitate the establishment of new populations of plant-associated microbes, including pathogens that can increase crop losses and food security concerns [1–3]. The repeated, inadvertent introduction of pathogens to plant production systems has the potential to cause genetic shifts in local and regional pathogen populations within each production cycle. Disease management strategies may include focusing on the most critical points of pathogen entry and spread, if knowledge of such routes within the system exists [4, 5]. Whole genome sequencing has enabled in-depth study of microbial populations in medical settings and surveillance of bacterial pathogens affecting human health [6–12]. For phytopathogenic bacteria, molecular epidemiological studies utilizing whole genome sequences have begun to shed light on pathogen evolution, geographic origins, and dissemination [13–19]. Genomic variation of bacterial plant pathogens can also be used to decipher pathogen movement in complex agricultural production systems [19] and the role of production systems in structuring regional populations.

*Xanthomonas perforans* (*Xp*) is one of four species that causes bacterial spot of tomato and pepper, which can result in major losses for growers via fruit spotting and foliar blighting [20–24]. Following its first report in 1991 in Florida, it quickly replaced *X. euvesicatoria* as the predominant cause of bacterial spot [25]. *Xp* is now broadly distributed on tomato throughout the world [21, 26]. Florida has been a focus of research on *Xp*, and is one of the top fresh market tomato producers in the United States [27]. A progression of studies looking at *Xp* populations since that time has revealed the diversity and change in type III secretion system effector content over time despite lack of any commercially deployed resistant cultivars [23, 28–32]. These results suggest that the complex tomato production system may be contributing to the diversity and structure of *Xp* populations.

Bacterial spot epidemics caused by *Xp* occur annually in Florida and other eastern states, but little is known about the source of inoculum each season [33–36]. To associate genetic variation in local pathogen populations with components of the tomato production system, we isolated and characterized 585 pathogenic *Xp* strains from 70 commercial tomato fields located in Florida and southern Georgia during the fall 2017 production season [28]. For each plant from which we isolated a strain, we collected metadata, including the specific farm and grower operation (each independent grower operation may consist of two or more separate farms), the geographic region (i.e., the county) of the farm, the transplant facility where the plant was initially sown and grown, and the specific tomato cultivar and seed producer. Sequencing of amplified portions of two genes (*mldB* and *maf*) allowed us to identify presence of all three previously reported *Xp* sequence types. Non-metric multidimensional scaling and network analyses using phenotypic and genotypic traits (including sequence type, streptomycin resistance, bacteriocin production, tomato race, and presence/absence of type III secretion system effectors) enabled us to associate sequence types with tomato production system variables, including farms and transplant facilities, to a limited extent. However, similar characterization profiles across strains hampered our ability to evaluate the association of strain genotypes to tomato production system variables. We hypothesized that using whole genome sequences from the fall 2017 collection would enable us to associate tomato production system variables with *Xp* population structure.

In this study, we analyzed 281 new *Xp* genomes from our fall 2017 collection, all isolated within a single Florida production season [28], to identify variables that structure the population genetic variation of *Xp* across the Florida tomato production system. We were specifically interested in whether specific pathogen genotypes were associated with specific sources (e.g., seed producers, transplant facilities, grower operations, farms, and geographic regions), which would identify possible routes for introduction and movement of new pathogen genotypes. Indeed, genome sequencing uncovered a novel genotype associated with multiple farms. We also found association of multiple components of the production system with pathogen population structure. Altogether, our study shows that the complexity of agricultural production systems is reflected in local microbial populations.

## Materials and methods

### Bacterial strains and growth conditions

A total of 281 bacterial strains were selected from a previous study of 585 *Xp* strains isolated from tomato tissue across Florida and southern Georgia production fields during the fall 2017 growing season [28]. For this study, we selected all strains collected from the five tomato cultivars that had the highest number of associated strains and fields. Supplementary Table S1 contains a list with metadata regarding the plant from which each strain was isolated. Bacterial strains stored at −80 °C in nutrient broth (BBL; Becton Dickinson and Co, Franklin Lakes, NJ, USA) with 30% glycerol were removed and streaked on nutrient agar, then incubated at 28 °C for 3-4 days to confirm appearance of pure cultures. For DNA extraction, cells were removed from nutrient agar plates and grown overnight in nutrient broth at 28 °C with shaking.

### DNA extraction and sequencing

Overnight nutrient broth cultures were subjected to DNA extraction using the Gram-negative bacterial DNA extraction protocol from the Wizard genomic DNA purification kit (Promega, Madison, WI). DNA extractions were submitted to the Microbial Genome Sequencing Center (MIGS; Pittsburgh, PA, USA) for library construction and sequencing. MIGS constructed libraries using methods described by Baym et al. [37], utilizing the Illumina Nextera kit (Illumina Inc., San Diego, CA, USA). Genomes were sequenced using the Illumina NextSeq 550 platform, providing 151-bp paired-end reads.

### Computational analyses

Unless specified otherwise, all genomic data processing was completed via shell scripts run on the University of Florida HiPerGator supercomputer. One reference strain from each of the three previously reported phylogenetic groups of Florida-isolated *Xp* was included in all analyses: 1: 91-118 (GCA_000192045.3), 2: Xp2010 (SAMN16406455), 3: Xp17-12 (SAMN16406456) [30, 35, 38, 39].

### Genome assembly

Draft genomes were assembled de novo using modified pipelines described in Timilsina et al. [30]. Adapters were removed from raw FASTQ reads and reads were paired using Trim Galore! (v. 0.6.3) with default parameters. Paired reads were assembled into contigs with SPAdes (v. 3.10.1) [40]. K-mers 21, 33, 55, 77, 99, and 127 were run and contigs that were smaller than 500-bp in length with a k-mer coverage of less than 2.0 were removed. Validated reads were aligned against the filtered contigs and output as a SAM-formatted alignment with default parameters of Bowtie 2 (v. 2.3.3) [41]. SAM files were converted to BAM files with SAMtools (v. 1.9) [42]. Draft assemblies were polished with default parameters of Pilon (v. 1.22) [43]. Genome assemblies were assessed for completeness and contamination with CheckM (v. 1.1.2) [44], using the *Xanthomonas* genus-level taxonomic marker set.

### Data availability of genomes and corresponding annotations

Assembled whole genome sequences and raw read data were deposited in the NCBI GenBank database under BioProject number PRJNA668343. Assembled genomes were annotated using the Department of Energy Joint Genome Institute’s Integrated Microbial Genomes (IMG) and Microbiomes annotation pipeline (v. 5.0.3) [45]. Specific accession numbers and genome identifiers for IMG and NCBI are provided in Supplementary Table S2.

### Core gene identification, alignment, and cluster analyses

Assembled genomes were annotated using default parameters for bacteria with Prokka (v. 1.10) [46]. Annotated genomes were analyzed with Roary (v. 3.12.0), specifying Roary to designate core genes as those with a 75% minimum percentage identity for BLASTp and present in all genomes, and using MAFFT [47] for performing the nucleotide alignment. The resulting nucleotide alignment file was converted to FASTA format for downstream programs using the seqret tool from EMBOSS [48]. ModelTest-NG [49] was used with default parameters to determine the appropriate substitution model for phylogenetic analyses. Based on both the Bayesian information criterion and the corrected Akaike information criterion, the general time-reversible substitution model with optimization of substitution rates across sites and estimation of invariable site proportions (GTR + I + G) had the best fit out of 88 DNA models tested. Phylogenetic analyses were performed with RAxML (v. 8.2.10) [50] using the pre-determined substitution model (‘GTRGAMMAI’), specifying 1000 rapid bootstraps [51]. The best-scoring multilocus tree was corrected to account for recombination with ClonalFrameML (v. 1.0) [52] using 1000 pseudo-bootstrap replicates. The core gene single nucleotide polymorphisms (SNPs) that were used to create the ClonalFrameML multilocus tree were analyzed with Rhierbaps (v. 1.1.3) [53, 54] with one level of clustering to assign strains to core gene clusters. iTOL (v. 5.6.3) [55] was used to visualize the multilocus trees generated with RAxML and ClonalFrameML, overlaid with colors indicating Rhierbaps cluster assignments.

### Network visualization

We evaluated how core gene clusters were partitioned across production system variables transplant facility, farm, and field. A related analysis, focusing on geographic location, was implemented for the network across the hierarchy of region, county, farm, and field variables. The R package igraph [56] and customized R [57] scripts were used to visualize the core gene cluster partitioning in relation to the plant history variables for all 281 strains. Links between hierarchical variable categories were constructed based on the observed production system paths for distribution of plant materials.

### Identification of SNPs

SNPs across genomes within clusters 2, 3, 4, and 5, as defined by the core gene clustering analysis, were identified based upon the protocol described by Abrahamian et al. [35], with modifications. SNP analyses were not performed for clusters 1 and 6, as they contained 10 strains each, which was too few to conduct population differentiation analyses. Illumina raw reads were paired with Geneious Prime (v. 2020.1.2; https://www.geneious.com) then trimmed using Trim Galore! (v. 0.6.3) with default parameters. Before alignment, the completed chromosome of each reference sequence was indexed using default parameters for the Burrows-Wheeler Aligner (BWA; v. 0.7.17) [58]. Plasmid sequences, as annotated in the reference strains, were excluded, because plasmids may be transmitted in local fields and not reflect production system variables [59]. Reads for each strain were aligned against the respective core gene cluster reference strain or closest relative (Xp2010 for cluster 2, Xp17-12 for cluster 3, and 91-118 for clusters 4 and 5) with the BWA-MEM algorithm [60], using default parameters. The SAM file outputs were converted to BAM files and sorted and indexed using SAMtools (v. 1.9) [42]. MarkDuplicates, within Picard [61], was used to remove duplicate raw reads caused by library construction artifacts from BAM files. SNPs were assigned using Freebayes (v. 1.3.1) [62], specifying a haploid genome, a requirement of at least eight supporting observations to consider the nucleotide a variant, and removing all insertions or deletions (indels), multi-nucleotide polymorphisms (MNPs), and complex allele observations (composite insertion and substitution events) from input. The VCF file output from Freebayes was filtered with the “VCFfilter” tool, part of the VCFlib [63] module, to remove all SNPs with a Phred score less than 50 (99.999% accuracy). VCF files for all strains within each core gene cluster were compressed with “bgzip”, indexed with Tabix [64], then merged with “VCF-merge” from VCFtools [65]. Strains JK3-3, JK22-5, JK37-1, JK38-1, JK45-2, JK46-3, JK52-4, and JK56-1 represented singletons, in that each strain was the only representative of its designated core gene cluster for a given production variable (e.g., for a particular farm or transplant facility). These singletons could not be used in statistical analyses, and thus were not included in the SNP analysis and were removed prior to VCF merging. The uncompressed merged VCF file was filtered with “VCFfilter” to remove all SNP positions with more than one alternate allele so that all variant positions were biallelic for downstream analyses. Three SNP positions within cluster 2 and two SNP positions within cluster 3 were removed; all SNPs within cluster 4 and 5 were biallelic. SNPs were annotated with SnpEff (v. 5.0) [66] using each core gene cluster’s corresponding reference strain sequence as the reference database. In total, 128 strains for cluster 2, 58 strains for cluster 3, 39 strains for cluster 4, and 28 strains for cluster 5, were included in the SNP analyses.

### Population structure

The merged VCF files for core gene clusters 2, 3, 4, and 5 were processed separately with the following protocol. Each VCF file was imported into R (v. 3.6.2) [67], within RStudio (v. 1.1.419) [68], using the vcfR package (v. 1.10.0) [69]. The periods, denoting nucleotides identical to the reference sequence within the VCF object, were each replaced with a zero, as required by the adegenet package [70]. The VCF object was converted into a genlight object with the adegenet package (v. 2.1.2) [70]. The Poppr package (v. 2.8.3) [71] was used to define population within each stratification (‘transplant facility’, ‘grower operation’, ‘region’, ‘county’, ‘farm’, ‘seed producer’, ‘cultivar’) for each strain. To determine the appropriate number of principal components (PCs) for discriminant analysis of principal components (DAPC), the “xvalDapc” command from the adegenet package was used. Once a range of PCs with the highest proportions of successful outcome prediction was determined, this was narrowed to a single PC value by running the same command for each whole number with 1000 replicates each, then selecting the PC with the highest mean proportions of successful outcome prediction (2: 26 PCs, 3: 12 PCs, 4: 6 PCs, 5: 2 PCs), as advised by Grünwald et al. [72]. Using Poppr [71], the population stratification was set prior to each DAPC run. DAPC was run using the adegenet package [70] in the genlight object, with the appropriate number of PCs and predefined population, then visualized with scatter plots. Population membership probability for each of the predetermined populations, which was inherently calculated as part of the DAPC object, was visualized with a composite stacked bar plot using ggplot2 (v. 3.3.2) [73], as advised by Tabima et al. [74]. To further quantify population structure, analysis of molecular variance (AMOVA) was performed using Poppr [71]. The population stratification was set prior to each AMOVA test. Significance tests using the ade4 package [75] were performed to determine if values were significantly different via 1000 random permutations of the sample matrices, as advised by Kamvar et al. [76].

### Genomic diversity analyses of SNPs

Nucleotide diversity within populations (π) and nucleotide substitutions per site among populations (D_xy_) were determined for each population by each stratification. The merged VCF file for each cluster, along with the corresponding population assignments, were imported into DnaSP (v. 6) [77] as a “multi-MSA data file analysis”. ModelTest-NG [49] was used with default parameters to determine the proper distance model to calculate F_ST_. To convert the merged VCF files into a compatible file type for ModelTest-NG to read, variants within each VCF file were concatenated with the appropriate reference sequence in Geneious Prime (v. 2020.1.2) and exported in FASTA format. PGDSpider (v. 2.1.1.5) [78] was used to convert the FASTA-formatted SNP files into Arlequin file format for each cluster and population stratification combination. Variance in allele frequencies among populations (F_ST_) was determined with Arlequin (v. 3.5) [79], using the Tamura distance model to calculate genetic distances between haplotypes, and significance of F_ST_ values (alpha = 0.05) was determined using 10,000 permutations of pairwise differences. Results of SNP genomic diversity analyses were manually combined into matrix format within Microsoft Excel.

## Results

### Strain collection and genome sequencing

In total, whole genomes of 281 strains, each collected from a different plant, from 35 fields on fifteen farms were sequenced. The plants represented 8 transplant facilities, 5 cultivars, 4 seed producers, 11 grower operations, and 8 counties. Supplementary Table S2 summarizes genome size, contig number, N50, coverage, GC content, completeness, contamination, and NCBI and IMG online database accessions for all sequenced strains. De novo assembled genomes averaged 51 contigs (range: 30–103) with an average coverage depth of 74.9X (range: 26.3X to 264.2X). GC content varied from 64.4 to 64.7%. Genome sizes ranged from 5.10 to 5.45 Mb. CheckM identified an average of 99.9% genome completeness, with the lowest value at 98.5%, and an average contamination of 0.84%, with the highest value at 1.78%.

### Phylogenetic analyses and cluster identification with the core genome

**Fig. 1:**
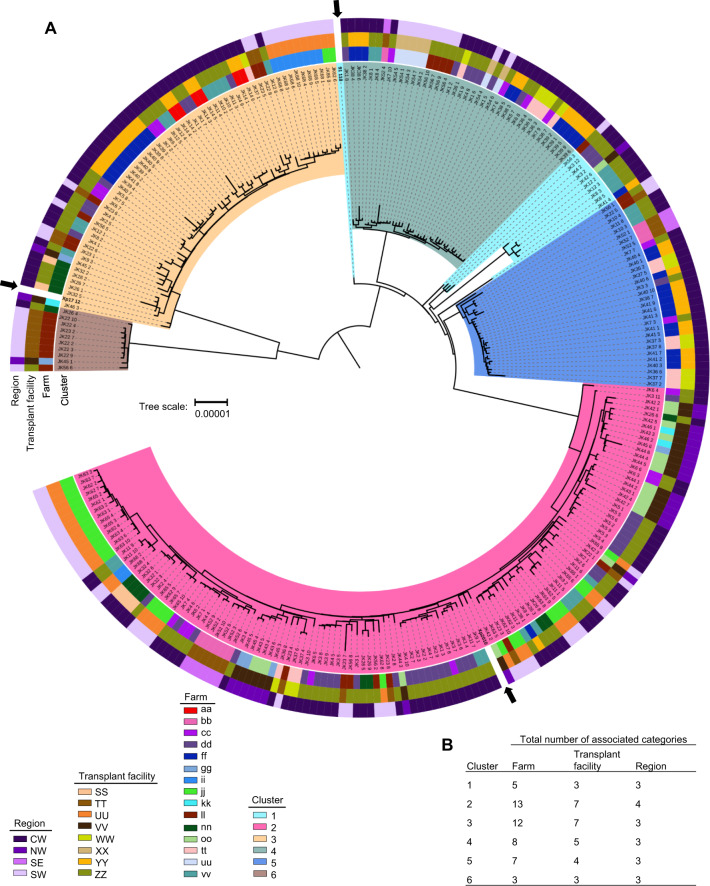
Phylogenetic tree of *Xanthomonas perforans* based on genetic distance of core gene SNPs, corrected for recombination. Clades are highlighted by their respective core gene cluster, as determined by Rhierbaps (**A**). Core gene cluster identity is denoted by highlights overlaid on clades. Association with the tomato production system variables farm, transplant facility, and region, for the plant each strain was isolated from, are indicated by different colored blocks within each respective ring surrounding the phylogenetic tree. Reference genomes from three previously reported Florida *X. perforans* strains are denoted with bold text and black arrows. The number of categories within each variable was summed for each of the six clusters (**B**).

Roary identified 3735 core genes across all sampled and three reference genomes, out of 6561 total genes annotated by Prokka. Of the 2826 accessory genes, 192 were present in at least 95% of strains but fewer than 100%, 957 were present in at least 15% of strains but fewer than 95%, and 1677 were present in at least one strain but fewer than 15%. Phylogenetic analyses of core gene SNPs grouped strains into several clades with greater than 99% bootstrap support (Supplementary Fig. S1). These clades remained after correcting for recombination with ClonalFrameML (Fig. 1). The mean relative rate of recombination (R/θ), estimated by ClonalFrameML, was 0.827, the average length of recombined fragments (δ) was 472 bp, and the average divergence between donor and recipient (ν) was 0.0193. Rhierbaps identified six distinct clusters present in the population (Fig. 1 and Supplementary Table S2). Of the 281 strains, 10 strains belonged to cluster 1, 129 to cluster 2, 61 to cluster 3, 40 to cluster 4, 31 to cluster 5, and 10 to cluster 6. Cluster 1 was polyphyletic and contained reference strain 91-118, although this strain was distinct from other cluster 1 strains. Strain 91-118 was also the reference strain closest to clusters 4 and 5. Cluster 2 contained reference strain Xp2010. Cluster 6 was most closely related to cluster 3, which contained reference strain Xp17-12. Addition of tomato production system variables to the core gene phylogenetic tree revealed that a diversity of farms, transplant facilities, and regions were represented within each cluster of strains (Fig. 1).

### Tomato production system network

**Fig. 2:**
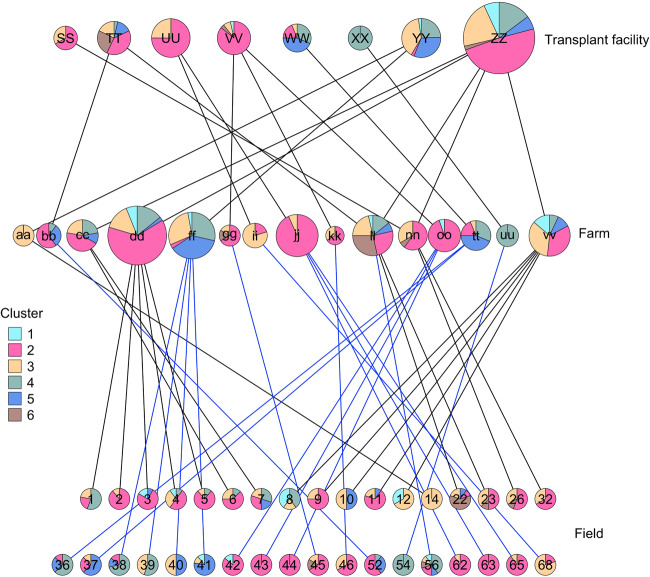
Network showing the distribution of core gene clusters across the tomato production system for variables transplant facility, farm, and field. Nodes (in rows) represent categories for each variable and links indicate hierarchical associations. All 281 strains from the collection are represented for each variable. Node size is proportional to the number of strains evaluated for a category, and the pie chart indicates the proportion of each core gene cluster. Black versus blue links from farms to fields distinguish fields that are in the top and bottom row, respectively.

The fresh-market tomato industry is complex, as demonstrated in hierarchical networks that show relationships of plants and sampled fields to the other tomato production system variables (farm, transplant facility: Fig. 2; farm, county, region: Supplementary Fig. S2; Supplementary Table S3). For example, we sampled plants on five farms that were seeded by transplant facility ‘ZZ’ (Fig. 2).

Placement of core gene clusters on the networks showed that clusters were distributed across plant sources. In one case, all strains associated with transplant facility ‘XX’ represented a single core gene cluster, and two farms contained only a single cluster (‘aa’ and ‘uu’), but these were the exceptions (Fig. 2). Overall, 40% of farms and 62.5% of transplant facilities (Fig. 2), as well as all cultivars sampled (Supplementary Table S3), were associated with four or more clusters. Geographically, all strains represented a single core gene cluster in only one county (‘f’, 6 strains from 1 field), strains from five or six clusters were found in each of three counties (‘a’, 45 strains from 5 fields; ‘c’, 49 strains from 5 fields; ‘g’, 113 strains from 15 fields), and at a larger scale, strains from three or more clusters were isolated from all four commercial tomato production regions (Supplementary Fig. S2). Despite collecting only 3–12 strains per field, three or more clusters were identified in 51.4% of the 35 fields sampled.

### SNP statistics based on the respective cluster reference strain

To examine population structure associated with tomato production system variables, given the genetic divergence among clusters, chromosomal SNPs were determined separately for core gene clusters 2, 3, 4, and 5 using the closest reference strain based on the core gene phylogenetic tree. In total, 1655 variant sites were identified across cluster 2 strains, 761 across cluster 3, 1823 across cluster 4, and 1624 across cluster 5 when compared to the reference strains. Clusters 4 and 5 had relatively more variant sites than clusters 2 and 3 (Table 1 and Supplementary Tables S4–7), which was expected as the reference strain, 91-118, used for clusters 4 and 5 was assigned to a different core gene cluster. When invariant sites within our sample were removed, there remained 1652, 691, 558, and 585 SNPs among strains within clusters 2, 3, 4, and 5, respectively.

**Table 1:** SNP annotation summaries based upon comparisons with reference strains Xp2010, Xp17-12, and 91-118 for core gene clusters 2, 3, and 4 and 5, respectively.

						Nonsynonymous		
Cluster	Number of strains		Total	Intragenic	Synonymous	Missense	Nonsense	Nonstop
2	128	Average	95	56	55	27	0	0
		Minimum	14	12	3	7	0	0
		Maximum	674	182	488	119	2	0
3	58	Average	127	126	86	26	1	0
		Minimum	94	93	61	18	0	0
		Maximum	359	358	266	59	3	0
4	39	Average	1486	1439	543	676	19	2
		Minimum	1400	1353	524	635	18	1
		Maximum	1526	1482	554	697	21	2
5	28	Average	1291	1248	508	580	20	6
		Minimum	1166	1128	457	521	18	6
		Maximum	1339	1296	524	602	22	6

### Population differentiation across tomato production system variables

**Fig. 3:**
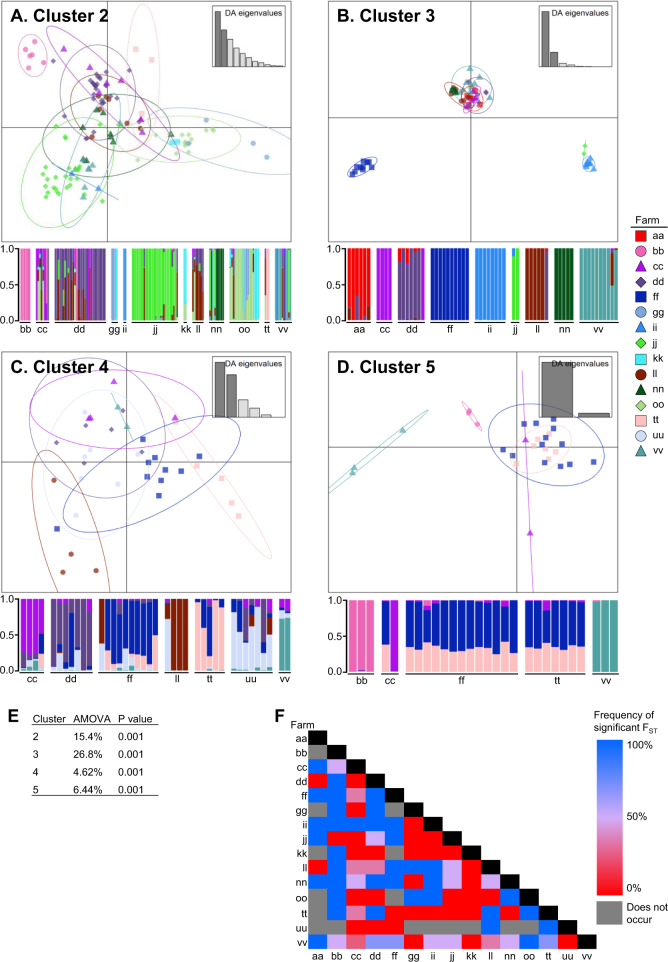
Population differentiation of *Xanthomonas perforans* across tomato production farms. Differentiation was based on chromosomal SNPs compared to the respective reference genome (Xp2010 for cluster 2, Xp17-12 for cluster 3, and 91-118 for clusters 4 and 5). Subfigures **A**–**D** depict subdivision of all farms according to discriminant analyses of principal components (DAPC) and associated population membership probabilities across clusters 2–5, with 26, 12, 6, and 2 principal components, respectively. Points and bars on DAPC plots and corresponding population membership probability plots, respectively, represent individual strains. DAPC plot points are surrounded by 95% inertia ellipses, and colors and shapes denote farm origin. Analysis of molecular variants (AMOVA) was calculated for samples within each DAPC plot (**E**). Variance in allele frequencies among populations (i.e., F_ST_) was calculated using the Tamura distance model. Matrix colors depict the frequency of a significant F_ST_ value (alpha = 0.05) across all specific farm pairwise occurrences for core gene clusters 2, 3, 4, and 5 (**F**). Supplementary Figs. S8, S9, S10, and S11 depict the same population differentiation graphics but are presented by core gene cluster.

**Fig. 4:**
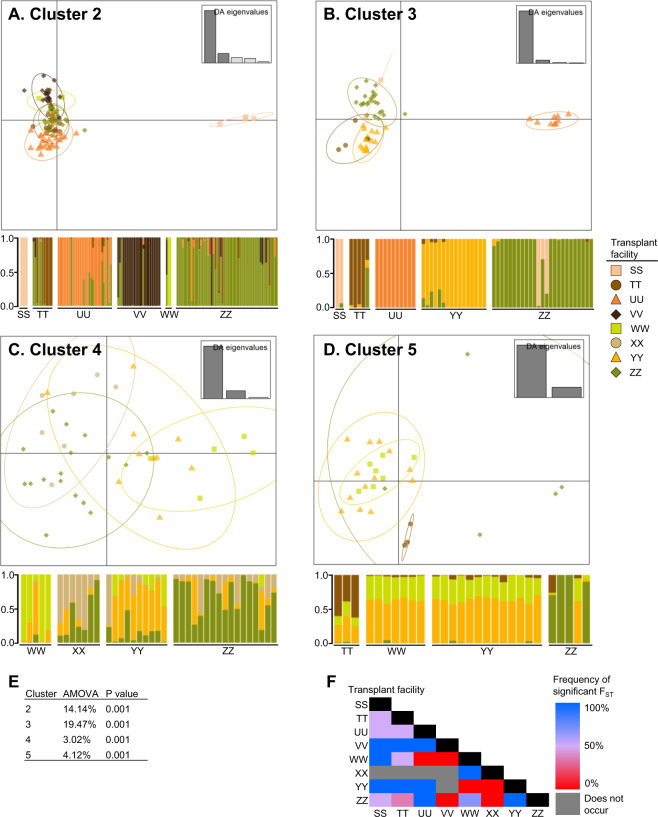
Population differentiation of *Xanthomonas perforans* across associated transplant facilities. Differentiation was based on chromosomal SNPs compared to the respective reference genome (Xp2010 for cluster 2, Xp17-12 for cluster 3, and 91-118 for clusters 4 and 5). Subfigures **A**–**D** depict subdivision of all transplant facilities according to discriminant analyses of principal components (DAPC) and associated population membership probabilities across clusters 2–5, with 26, 12, 6, and 2 principal components, respectively. Points and bars on DAPC plots and corresponding population membership probability plots, respectively, represent individual strains. DAPC plot points are surrounded by 95% inertia ellipses, and colors and shapes denote transplant facility association. Analysis of molecular variants (AMOVA) was calculated for each core gene cluster (**E**). Variance in allele frequencies among populations (i.e., F_ST_) was calculated using the Tamura distance model. Matrix colors depict the frequency of a significant F_ST_ value (alpha = 0.05) across all specific transplant facility pairwise occurrences for core gene clusters 2, 3, 4, and 5 (**F**). Supplementary Figs. S8, S9, S10, and S11 depict the same population differentiation graphics but are presented by core gene cluster.

Analyses of population structure within core gene clusters revealed significant genetic variation among farms and transplant facilities (Figs. 3 and 4). We also found genetically differentiated populations among regions, cultivars, counties, seed producers, and grower operations (Supplementary Figs. S3–7; presented by core gene cluster in Supplementary Figs. S8–11). In general, analysis of molecular variance indicated that populations defined by different farm and transplant facility categories were associated with more genetic variation in cluster 3 than cluster 2, and the least differentiated in clusters 4 and 5 (Figs. 3E and 4E). Nested analysis of molecular variance for the hierarchical variables farm, county, and region revealed most of the genetic variation was found within and between farms, except for cluster 5 which showed more variation among counties than farm (Supplementary Table S8). Pairwise genetic diversity statistics for categories within each variable showed that approximately half of the pairwise comparisons among populations defined by the variable categories were significantly differentiated as measured by F_ST_ (Figs. 3F, 4F, Supplementary Figs. S3F, S4F, S5F, S6F, S7F, and Supplementary Tables S9–15). SNP variation within clusters 4 and 5 could be explained with only six and two principal components, respectively, and thus were generally not as informative for population differentiation compared to the other two analyzed clusters.

*Xp* populations associated with some farms and transplant facilities were clearly genetically distinct from other Florida strains as indicated by assignment of all strains within a given category (farm or transplant facility) to a distinct population using discriminant analysis of principal components (Figs. 3A–D and 4A–D). For example, cluster 3 strains collected from farm ‘ff’ were distinct from all other strains in cluster 3 (Fig. 3B and Supplementary Table S4). Strains from farms ‘ii’ and ‘jj’, which were associated with the same grower operation, transplant facility, region, and cultivar, both contained strains from only clusters 2 and 3, and strains from these farms were clustered in the core gene phylogenetic tree and DAPC analysis (Figs. 1 and 3). Meanwhile, the strains from these farms were genetically distinct from other cluster 3 strains and many of the cluster 2 strains (Fig. 3), which may be explained by both farms receiving plants from transplant facility ‘UU’. Farms ‘ii’ and ‘jj’ and transplant facility ‘UU’ were within a single grower operation that produced its own transplants. Cluster 3 strains associated with transplant facility ‘UU’ were especially genetically distinct from other strains within cluster 3 (Fig. 4).

Genetically similar strains were also collected from different farms, suggesting common sources. Farms ‘cc’ and ‘dd’ contained genetically similar strains in clusters 2 and 3 (Figs. 1A and 3A) and both farms obtained plants from transplant facility ‘ZZ’ (Fig. 2) but were located in different counties (Supplementary Fig. S2). In contrast, farms ‘ff’ and ‘tt’ contained genetically similar cluster 4 and 5 strains (Figs. 1A and 3C, D) and obtained plants from different transplant facilities, but were located within the same county. Farms ‘ll’ and ‘nn’ were in the same grower operation but located in different regions. They produced strains from six and three clusters respectively, and cluster 2 and 3 strains that were similar to strains from other farms (Fig. 3). Notably these farms were associated with multiple transplant facilities and cultivars. Within cluster 2, strains associated with transplant facility ‘SS’ were genetically differentiated from strains from other facilities; however, the only farm sampled that received plants from ‘SS’ was ‘nn’, which also received plants from ‘ZZ’ (Fig. 2). In Fig. 1, cluster 2 strains associated with ‘SS” were genetically similar to each other whereas the strains from ‘nn’ that were isolated from plants originating from ‘ZZ’ were more dispersed throughout cluster 2.

## Discussion

The ability to rapidly sequence whole genomes has recently enabled plant bacteriologists to link environmental factors and agricultural production practices with pathogen genetics to better understand pathogen emergence and dissemination [13–16, 35]. Typically, such studies have focused efforts on bacterial strains collected over time and over larger geographic scales. In this study, we examined 281 strains collected in a specific geographic region over a single production cycle to provide insight into the genetic diversity and pathogen dissemination that occurs at local scales. Consequently, we uncovered previously unknown genetic variation in *Xp* in Florida tomato production. Through our in-depth analysis of *Xp* within a complex agricultural production system, we identified genetically distinct populations among farms and grower operations, but also genetically similar strains among farms that received plants from the same transplant facilities. These results suggest that strain movement is largely occurring within farms and via transplants. We note that all strains were isolated from tomato production fields and we used the known history of the tomato plant from which each strain was isolated to evaluate associations across the production system.

We identified *Xp* population structure across each variable of the tomato production system, including farm, transplant facility, cultivar, county, region, seed producer, and grower operation. These results suggest that each of these variables contributes, on their own or via correlation with other variables, to the genetic composition of the pathogen in tomato fields. In many cases, pairwise population differentiation between categories of system variables was consistent across core gene clusters, suggesting shared mechanisms governing strain movement and dispersal. However, we also observed varying levels of differentiation across variable categories among core gene clusters, and genetic differentiation among farms or transplant facilities that was not consistent between clusters. Our results suggest strains in different core gene clusters may move differentially throughout the tomato production system. This finding is similar to that from large-scale epidemiological studies of bacterial pathogens associated with human health which have suggested differential dispersal across genetic clusters for various species [6, 8, 11]. For example, some *Xp* clusters may be present in the local environment and disperse among fields, whereas others may be introduced to fields only via transplants, thus their population structure is influenced by their existing distribution and perhaps their fitness in the environment. We observed cluster 3 strains were genetically distinct across categories for most variables. We speculate that cluster 3, which is a more recently identified lineage of *Xp* in Florida, has had less time to move throughout the Florida tomato production system than clusters that emerged earlier.

Strains associated with some farms and transplant facilities represented a distinct *Xp* population, while many farms and transplant facilities were not associated with a distinct population of strains. As illustrated in Fig. 2 and even more so in our larger survey [28], many transplant facilities simultaneously grow plants for different farms. Likewise, commercial farms grow a variety of cultivars from different seed lots, which may have originated from multiple transplant facilities. At any given time, a single transplant facility or farm can contain hundreds of thousands of plants from a variety of seed producers and/or seed lots [36]. *Xp* may spread within or between farms or transplant facilities via workers, equipment, wind, rain, irrigation, weeds, or plant debris. Abrahamian et al. [36] demonstrated *Xp* moves rapidly, and often asymptomatically, within transplant facilities via aerosols produced from overhead irrigation. When plants are produced within a transplant facility or on a farm that contains a limited number of seed lots or cultivars at one time, and movement of potentially contaminated people or equipment between transplant facilities or fields is restricted, this could reduce the number of pathogen genotypes introduced to a given set of plants. The use of this production strategy by some growers may explain why some farms and transplant facilities were associated with less genetically diverse populations. Abrahamian et al. [35] studied strains from two growers who produced their own tomato transplants and found that strains isolated from transplant facilities and fields were more similar within a given grower operation than between operations. However, the study was conducted on a limited scale with grower operations that produce their own transplants, whereas many growers do not produce their own transplants, so it was unknown whether this finding was consistent across the tomato production system. Indeed, we found a similar pattern of strain clustering within farms and particularly within grower operations that produce their own transplants. Many Florida growers opt to instead outsource to transplant producers who grow transplants for multiple growers. We found that farms that outsourced their transplant production tended to produce diverse populations of strains that resembled populations from other farms. Previous studies in the 1980s and 1990s with *X. euvesicatoria* showed that volunteer tomato plants and crop residue [33] or seeds [34] were the primary inoculum source, but similar studies have not been conducted with *X. perforans*.

Some closely related strains came from plants that did not have common variable categories across any of the examined tomato production system variables. For example, strains within the monophyletic cluster 6 were associated with three distinct plant histories that did not share any common production system categories, nor were they isolated from the same production region. This finding is similar to that of whole genome-based studies of *Salmonella* Typhimurium by Mather et al. [10] and Mellor et al. [12], where authors were unable to associate diverse genotypes with variables that were previously thought to be critical for pathogen transmission. During our study, Hurricane Irma moved northward throughout the length of the Florida peninsula and caused some producers to preemptively move transplants out of the hurricane’s path, which may have facilitated long-distance pathogen movement and mixing of plants from different transplant facilities [28]. As a newly described genotype, we expect that future studies will illuminate the origins and dissemination of cluster 6 strains.

Sequencing whole genomes of a much larger number of strains than in previous studies allowed us to detect novel lineages. We identified six *Xp* core gene clusters in the population, whereas only three clusters have been identified in previous Florida-based genomic studies [30, 35, 39]. In our original study of 585 strains from this collection using MLSA of two loci, *Xp* strains that grouped within clusters 4 and 5 were lumped with cluster 1 strains, and cluster 6 strains were identified as cluster 3 strains [28]. Newberry et al. [80] also identified two novel *Xp* genetic clusters associated with tomatoes and peppers in Alabama via sequencing of only eight strains. Identification of two new clusters within a small sample size was proposed to be indicative of greater diversity within the Alabama population. Our study also reveals changing genetic diversity and low frequency variants in Florida *Xp* populations. In recent studies, genetic diversity in *Xp* has been associated with variation in Type III secreted effectors and other genes important in the plant-pathogen interaction [30, 80, 81], suggesting that strains in different clusters could have different relative fitness on plants or even in different production environments (e.g., seeds, transplant facilities, and open fields). MLSA has traditionally been used to assess population structure and variation, and to monitor populations for introductions of new genotypes. Based on our findings of an expanding number of genetically distinct groups of strains in Florida tomato production, and our knowledge that *Xp* readily evolves via recombination [30, 80, 81], genome sequencing will be important for continued monitoring of ecologically and epidemiologically relevant variation in *Xp*.

While our robust collection allowed us to examine the connectedness across many variables within the tomato production system involving plant material movement and field location, our ability to infer the point of strain introduction into the production system was limited by the complexity of the system and our decision to take samples from field plants at a single time point per field. Thus, further studies focused on pathogen spread are needed to understand strain-specific movement within and among production system variables. Overall, this work shows that the genetic variation of a bacterial plant pathogen is shaped by the structure of the plant production system. Further, our study shows that modifying plant production systems could limit the extent of pathogen diversity on plants in production fields.

## Supplementary information

Supplementary tables 1-15

Supplementary figures 1-11

## References

[1] Strange RN, Scott PR (2005). Plant disease: a threat to global food security. Annu Rev Phytopathol..

[2] Savary S, Willocquet L, Pethybridge SJ, Esker P, McRoberts N, Nelson A (2019). The global burden of pathogens and pests on major food crops. Nat Ecol Evol..

[3] Savary S, Bregaglio S, Willocquet L, Gustafson D, Mason D’Croz D, Sparks A (2017). Crop health and its global impacts on the components of food security. Food Secur..

[4] Garrett KA, Alcalá-Briseño RI, Andersen KF, Buddenhagen CE, Choudhury RA, Fulton JC (2018). Network analysis: a systems framework to address grand challenges in plant pathology. Annu Rev Phytopathol..

[5] Pautasso M, Xu X, Jeger MJ, Harwood TD, Moslonka-Lefebvre M, Pellis L (2010). Disease spread in small-size directed trade networks: the role of hierarchical categories. J Appl Ecol..

[6] Bryant JM, Grogono DM, Rodriguez-Rincon D, Everall I, Brown KP, Moreno P (2016). Emergence and spread of a human-transmissible multidrug-resistant nontuberculous mycobacterium. Science..

[7] Yang C, Zhang X, Fan H, Li Y, Hu Q, Yang R (2019). Genetic diversity, virulence factors and farm-to-table spread pattern of *Vibrio parahaemolyticus* food-associated isolates. Food Microbiol..

[8] Dallman TJ, Byrne L, Ashton PM, Cowley LA, Perry NT, Adak G (2015). Whole-genome sequencing for national surveillance of Shiga toxin-producing *Escherichia coli* O157. Clin Infect Dis..

[9] Kwong JC, Mercoulia K, Tomita T, Easton M, Li HY, Bulach DM (2016). Prospective whole-genome sequencing enhances national surveillance of *Listeria monocytogenes*. J Clin Microbiol..

[10] Mather AE, Reid SW, Maskell DJ, Parkhill J, Fookes MC, Harris SR (2013). Distinguishable epidemics of multidrug-resistant *Salmonella* Typhimurium DT104 in different hosts. Science..

[11] Richards VP, Velsko IM, Alam T, Zadoks RN, Manning SD, Pavinski Bitar PD (2019). Population gene introgression and high genome plasticity for the zoonotic pathogen *Streptococcus agalactiae*. Mol Biol Evol..

[12] Mellor KC, Petrovska L, Thomson NR, Harris K, Reid SWJ, Mather AE (2019). Antimicrobial resistance diversity suggestive of distinct *Salmonella* Typhimurium sources or selective pressures in food-production animals. Front Microbiol..

[13] Monteil CL, Yahara K, Studholme DJ, Mageiros L, Méric G, Swingle B (2016). Population-genomic insights into emergence, crop adaptation and dissemination of *Pseudomonas syringae* pathogens. Micro Genom..

[14] Perez-Quintero AL, Ortiz-Castro M, Lang JM, Rieux A, Wu G, Liu S (2020). Genomic acquisitions in emerging populations of *Xanthomonas vasicola* pv. *vasculorum* infecting corn in the United States and Argentina. Phytopathology..

[15] McCann HC, Li L, Liu Y, Li D, Pan H, Zhong C (2017). Origin and evolution of the kiwifruit canker pandemic. Genome Biol Evol..

[16] Quibod IL, Atieza-Grande G, Oreiro EG, Palmos D, Nguyen MH, Coronejo ST (2020). The Green Revolution shaped the population structure of the rice pathogen *Xanthomonas oryzae* pv. *oryzae*. ISME J..

[17] Straub C, Colombi E, McCann H. Population genomics of bacterial plant pathogens. Phytopathology. 2021. 10.1094/PHYTO-09-20-0412-RVW. 10.1094/PHYTO-09-20-0412-RVW 33179999

[18] Vinatzer BA, Monteil CL, Clarke CR (2014). Harnessing population genomics to understand how bacterial pathogens emerge, adapt to crop hosts, and disseminate. Ann Rev Phytopathol..

[19] Weisberg AJ, Davis EW, Tabima JF, Belcher MS, Miller M, Kuo C (2020). Unexpected conservation and global transmission of agrobacterial virulence plasmids. Science..

[20] Jones JB, Lacy GH, Bouzar H, Stall RE, Schaad NW (2004). Reclassification of the xanthomonads associated with bacterial spot disease of tomato and pepper. Syst Appl Microbiol..

[21] Potnis N, Timilsina S, Strayer A, Shantharaj D, Barak JD, Paret ML (2015). Bacterial spot of tomato and pepper: diverse *Xanthomonas* species with a wide variety of virulence factors posing a worldwide challenge. Mol Plant Pathol..

[22] VanSickle J, Weldon R. The economic impact of bacterial leaf spot on the tomato industry. Tomato Inst Proc. 2009:30–31 https://plantpath.ifas.ufl.edu/rsol/RalstoniaPublications_PDF/Tomato_Institute_Proceedings_09.pdf.

[23] Horvath DM, Stall RE, Jones JB, Pauly MH, Vallad GE, Dahlbeck D (2012). Transgenic resistance confers effective field level control of bacterial spot disease in tomato. PLOS One..

[24] Kunwar S, Iriarte F, Fan Q, Evaristo da Silva E, Ritchie L, Nguyen NS (2018). Transgenic expression of *EFR* and *Bs2* genes for field management of bacterial wilt and bacterial spot of tomato. Phytopathology..

[25] Jones JB, Bouzar H, Somodi GC, Stall RE, Pernezny K, El-Morsy G (1998). Evidence for the preemptive nature of tomato race 3 of *Xanthomonas campestris* pv. *vesicatoria* in Florida. Phytopathology..

[26] Timilsina S, Jibrin MO, Potnis N, Minsavage GV, Kebede M, Schwartz A (2015). Multilocus sequence analysis of xanthomonads causing bacterial spot of tomato and pepper plants reveals strains generated by recombination among species and recent global spread of *Xanthomonas gardneri*. Appl Environ Microbiol..

[27] United States Department of Agriculture. National Agricultural Statistics Service. Washington, DC: United States Department of Agriculture; 2019.

[28] Klein-Gordon JM, Xing Y, Garrett KA, Abrahamian P, Paret ML, Minsavage GV, et al. Assessing changes and associations in the *Xanthomonas perforans* population across Florida commercial tomato fields via a state-wide survey. Phytopathology. 2021;111:1029–1041. 10.1094/PHYTO-09-20-0402-R 33048630

[29] Vallad GE, Timilsina S, Adkison H, Potnis N, Minsavage G, Jones J, et al. A recent survey of xanthomonads causing bacterial spot of tomato in Florida provides insights into management strategies. Tomato Inst Proc. 2013:25–27 https://swfrec.ifas.ufl.edu/docs/pdf/veghort/tomato-institute/proceedings/ti13_proceedings.pdf.

[30] Timilsina S, Pereira-Martin JA, Minsavage GV, Iruegas-Bocardo F, Abrahamian P, Potnis N (2019). Multiple recombination events drive the current genetic structure of *Xanthomonas perforans* in Florida. Front Microbiol..

[31] Burlakoti R, Hsu C, Chen J, Wang J (2018). Population dynamics of Xanthomonads associated with bacterial spot of tomato and pepper during twenty-seven years across Taiwan. Plant Dis..

[32] Araújo ER, Costa JR, Ferreira MASV, Quezada-Duval AM (2017). Widespread distribution of *Xanthomonas perforans* and limited presence of *X. gardneri* in Brazil. Plant Pathol..

[33] Jones JB, Pohronezny KL, Stall RE, Jones JP (1986). Survival of *Xanthomonas campestris* pv. *vesicatoria* in Florida on tomato crop residue, weeds, seeds, and volunteer tomato plants. Phytopathology..

[34] Sijam K, Chang CJ, Gitaitis RD (1991). An agar medium for the isolation and identification of *Xanthomonas campestris* pv. *vesicatoria* from seed. Phytopathology..

[35] Abrahamian P, Timilsina S, Minsavage GV, Potnis N, Jones JB, Goss EM (2019). Molecular epidemiology of *Xanthomonas perforans* outbreaks in tomato plants from transplant to field as determined by single-nucleotide polymorphism analysis. Appl Environ Microbiol..

[36] Abrahamian P, Sharma A, Jones J, Vallad GE. Dynamics and spread of bacterial spot epidemics in tomato transplants grown for field production. Plant Dis. 2021 in press. 10.1094/PDIS-05-20-0945-RE 32865478

[37] Baym M, Kryazhimskiy S, Lieberman TD, Chung H, Desai MM, Kishony R (2015). Inexpensive multiplexed library preparation for megabase-sized genomes. PLOS One..

[38] Tudor-Nelson SM, Minsavage GV, Stall RE, Jones JB (2003). Bacteriocin-like substances from tomato race 3 strains of *Xanthomonas campestris* pv. *vesicatoria*. Bacteriology..

[39] Schwartz A, Potnis N, Timilsina S, Wilson M, Patane J, Martins J (2015). Phylogenomics of *Xanthomonas* field strains infecting pepper and tomato reveals diversity in effector repertoires and identifies determinants of host specificity. Front Microbiol..

[40] Nurk S, Bankevich A, Antipov D, Gurevich AA, Korobeynikov A, Lapidus A (2013). Assembling single-cell genomes and mini-metagenomes from chimeric MDA products. J Comput Biol..

[41] Langmead B, Salzberg SL (2012). Fast gapped-read alignment with Bowtie 2. Nat Methods..

[42] Li H, Handsaker B, Wysoker A, Fennell T, Ruan J, Homer N (2009). The sequence alignment/map format and SAMtools. Bioinform..

[43] Walker BJ, Abeel T, Shea T, Priest M, Abouelliel A, Sakthikumar S (2014). Pilon: an integrated tool for comprehensive microbial variant detection and genome assembly improvement. PLOS One..

[44] Parks DH, Imelfort M, Skennerton CT, Hugenholtz P, Tyson GW (2015). CheckM: assessing the quality of microbial genomes recovered from isolates, single cells, and metagenomes. Genome Res..

[45] Chen IA, Chu K, Palaniappan K, Pillay M, Ratner A, Huang J (2019). IMG/M v.5.0: an integrated data management and comparative analysis system for microbial genomes and microbiomes. Nucleic Acids Res..

[46] Seemann T (2014). Prokka: rapid prokaryotic genome annotation. Bioinformatics..

[47] Katoh K, Standley DM (2013). MAFFT multiple sequence alignment software version 7: improvements in performance and usability. Mol Biol Evol..

[48] Rice P, Longden I, Bleasby A (2000). EMBOSS: the European molecular biology open software suite. Trends Genet..

[49] Darriba D, Posada D, Kozlov AM, Stamatakis A, Morel B, Flouri T (2019). ModelTest-NG: a new and scalable tool for the selection of DNA and protein evolutionary models. Mol Biol Evol..

[50] Stamatakis A (2014). RAxML version 8: a tool for phylogenetic analysis and post-analysis of large phylogenies. Bioinformatics..

[51] Stamatakis A, Hoover P, Rougemont J (2008). A rapid bootstrap algorithm for the RAxML web servers. Syst Biol..

[52] Didelot X, Wilson DJ (2015). ClonalFrameML: efficient inference of recombination in whole bacterial genomes. PLOS Comput Biol..

[53] Tonkin-Hill G, Lees JA, Bentley SD, Frost SDW, Corander J (2018). RhierBAPS: an R implementation of the population clustering algorithm hierBAPS. Wellcome Open Res..

[54] Cheng L, Connor TR, Sirén J, Aanensen DM, Corander J (2013). Hierarchical and spatially explicit clustering of DNA sequences with BAPS software. Mol Biol Evol..

[55] Letunic I, Bork P (2007). Interactive Tree Of Life (iTOL): an online tool for phylogenetic tree display and annotation. Bioinformatics..

[56] Csardi G, Nepusz T. The igraph software package for complex network research. 2006; InterJ., Complex Systems:1695.

[57] R Core Team. R: a language and environment for statistical computing. Vienna, Austria: R Foundation for Statistical Computing; 2020.

[58] Li H, Durbin R (2009). Fast and accurate short read alignment with Burrows-Wheeler transform. Bioinformatics..

[59] Canteros BI, Minsavage GV, Jones JB, Stall RE (1995). Diversity of plasmids in *Xanthomonas campestris* pv. *vesicatoria*. Phytopathology..

[60] Li H. Aligning sequence reads, clone sequences and assembly contigs with BWA-MEM. arXiv. 2013. https://arxiv.org/abs/1303.3997.

[61] Broad Institute: Picard. http://broadinstitute.github.io/picard/ 2019.

[62] Garrison E, Marth G. Haplotype-based variant detection from short-read sequencing. arXiv. 2012. https://arxiv.org/abs/1207.3907.

[63] Garrison, E, Kronenberg, ZN, Dawson, ET, Pedersen, BS, Prins, P. Vcflib and tools for processing the VCF variant call format. BioRxiv. 2021. 10.1371/journal.pcbi.1009123 PMC9286226 35639788

[64] Li H (2011). Tabix: fast retrieval of sequence features from generic TAB-delimited files. Bioinformatics..

[65] Danecek P, Auton A, Abecasis G, Albers CA, Banks E, DePristo MA (2011). The variant call format and VCFtools. Bioinformatics..

[66] Cingolani P, Platts A, Wang LL, Coon M, Nguyen T, Wang L (2012). A program for annotating and predicting the effects of single nucleotide polymorphisms, SnpEff. Fly..

[67] R Core Team. A language and environment for statistical computing. Vienna, Austria: R Foundation for Statistical Computing; 2019.

[68] RStudio Team. RStudio: Integrated Development for R. Boston, MA: RStudio Inc.; 2016.

[69] Knaus B, Grünwald NJ (2017). vcfR: a package to manipulate and visualize variant call format data in R. Mol Ecol Res..

[70] Jombart T (2008). adegenet: a R package for the multivariate analysis of genetic markers. Bioinform..

[71] Kamvar ZN, Tabima JF, Grünwald NJ (2014). Poppr: an R package for genetic analysis of populations with clonal, partially clonal, and/or sexual reproduction. PeerJ..

[72] Grünwald NJ, Kamvar ZN, Everhart SE. Population genetics and genomics in R: Discriminant analysis of principal components (DAPC). 2020. https://grunwaldlab.github.io/Population_Genetics_in_R/DAPC.html.

[73] Wickham H. ggplot2: elegant graphics for data analysis. New York: Springer-Verlag; 2016.

[74] Tabima JF, Knaus B, Grünwald NJ. Population genetics and genomics in R: GBS analysis. 2020. https://grunwaldlab.github.io/Population_Genetics_in_R/gbs_analysis.html.

[75] Dray S, Dufour A (2007). The ade4 package: implementing the duality diagram for ecologists. J Stat Softw..

[76] Kamvar ZN, Everhart SE, Grünwald NJ. Population genetics and genomics in R: AMOVA. 2020. https://grunwaldlab.github.io/Population_Genetics_in_R/AMOVA.html.

[77] Rozas J, Ferrer-Mata A, Sanchez-DelBarrio JC, Guirao-Rico S, Librado P, Ramos-Onsins SE (2017). DnaSP 6: DNA sequence polymorphism analysis of large data sets. Mol Biol Evol..

[78] Lischer HE, Excoffier L (2012). PGDSpider: an automated data conversion tool for connecting population genetics and genomics programs. Bioinformatics..

[79] Excoffier L, Lischer HEL (2010). Arlequin suite ver 3.5: a new series of programs to perform population genetics analyses under Linux and Windows. Mol Ecol Resour..

[80] Newberry EA, Bhandari R, Minsavage GV, Timilsina S, Jibrin MO, Kemble J, et al. Independent evolution with the gene flux originating from multiple *Xanthomonas* species explains genomic heterogeneity in *Xanthomonas perforans*. Appl Environ Microbiol. 2019;85:e00885–19. 10.1128/AEM.00885-19 PMC6805091 31375496

[81] Jibrin MO, Potnis N, Timilsina S, Minsavage GV, Vallad GE, Roberts PD, et al. Genomic inference of recombination-mediated evolution in *Xanthomonas euvesicatoria* and *X. perforans*. Appl Environ Microbiol. 2018; 84:e00136–18. 10.1128/AEM.00136-18 PMC6007113 29678917

