# Supplementary material for: Whole genome sequences reveal the Xanthomonas perforans population is shaped by the tomato production system
Source: ISME J. 2021 Sep 6;16(2):591–601. doi: 10.1038/s41396-021-01104-8 (PMC8776747; doi:10.1038/s41396-021-01104-8)
Supplement: Supplementary file 2 — Supplementary figures 1-11 [file 41396_2021_1104_MOESM2_ESM.pdf]

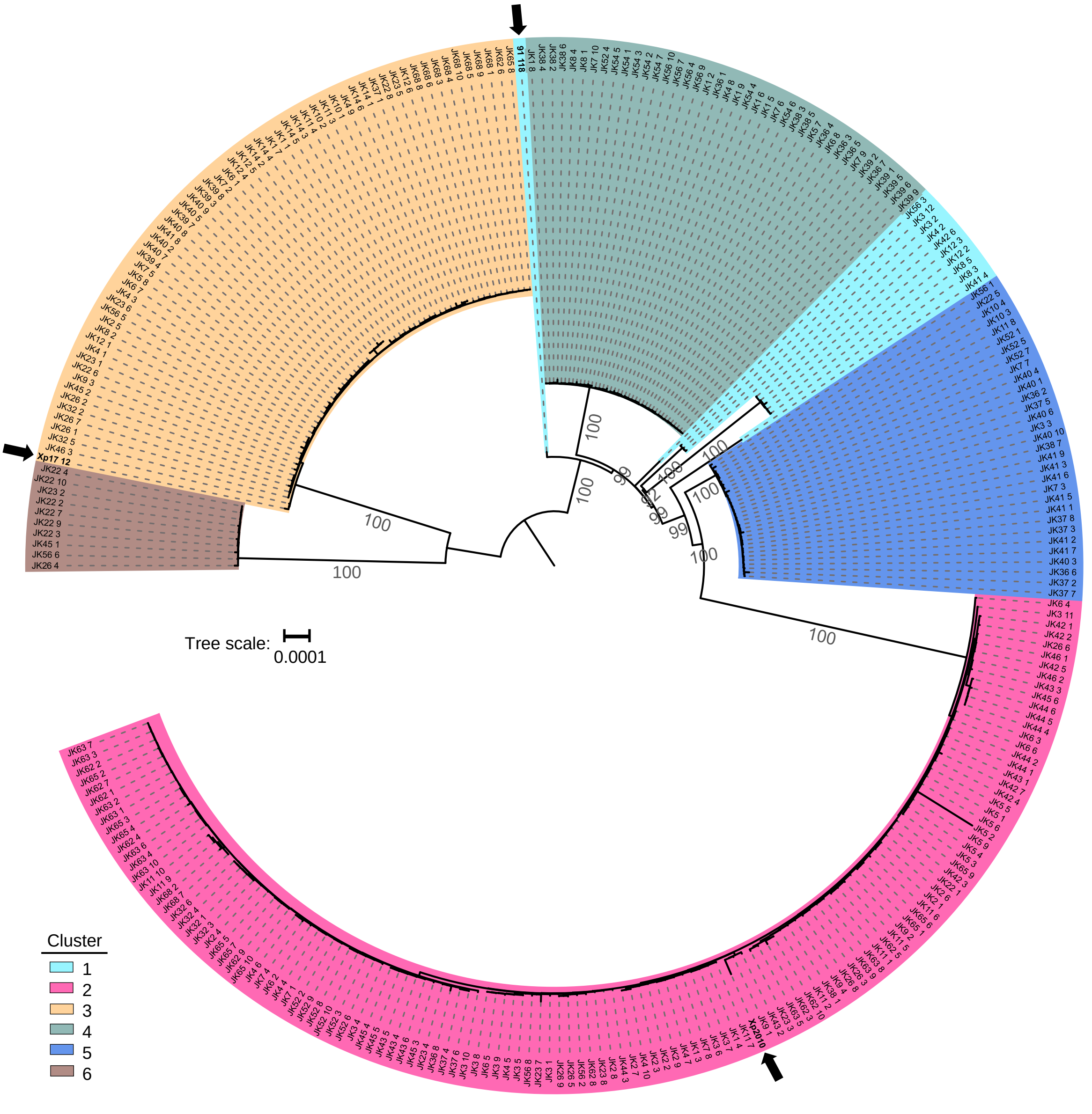

Supplementary figure 1. Phylogenetic tree of *Xanthomonas perforans* based on genetic distance of core gene SNPs. Core genes were called via Roary and phylogenetic analyses were performed with RAxML. One thousand rapid bootstraps were computed and the bootstrap support values distinguishing major branches between strain groupings are shown. Clades are highlighted by their respective core gene cluster, as determined by Rhierbaps. Cluster identity is denoted by highlights overlaid on clades. Reference genomes from three previously reported Florida *X. perforans* strains are denoted with bold text and black arrows.

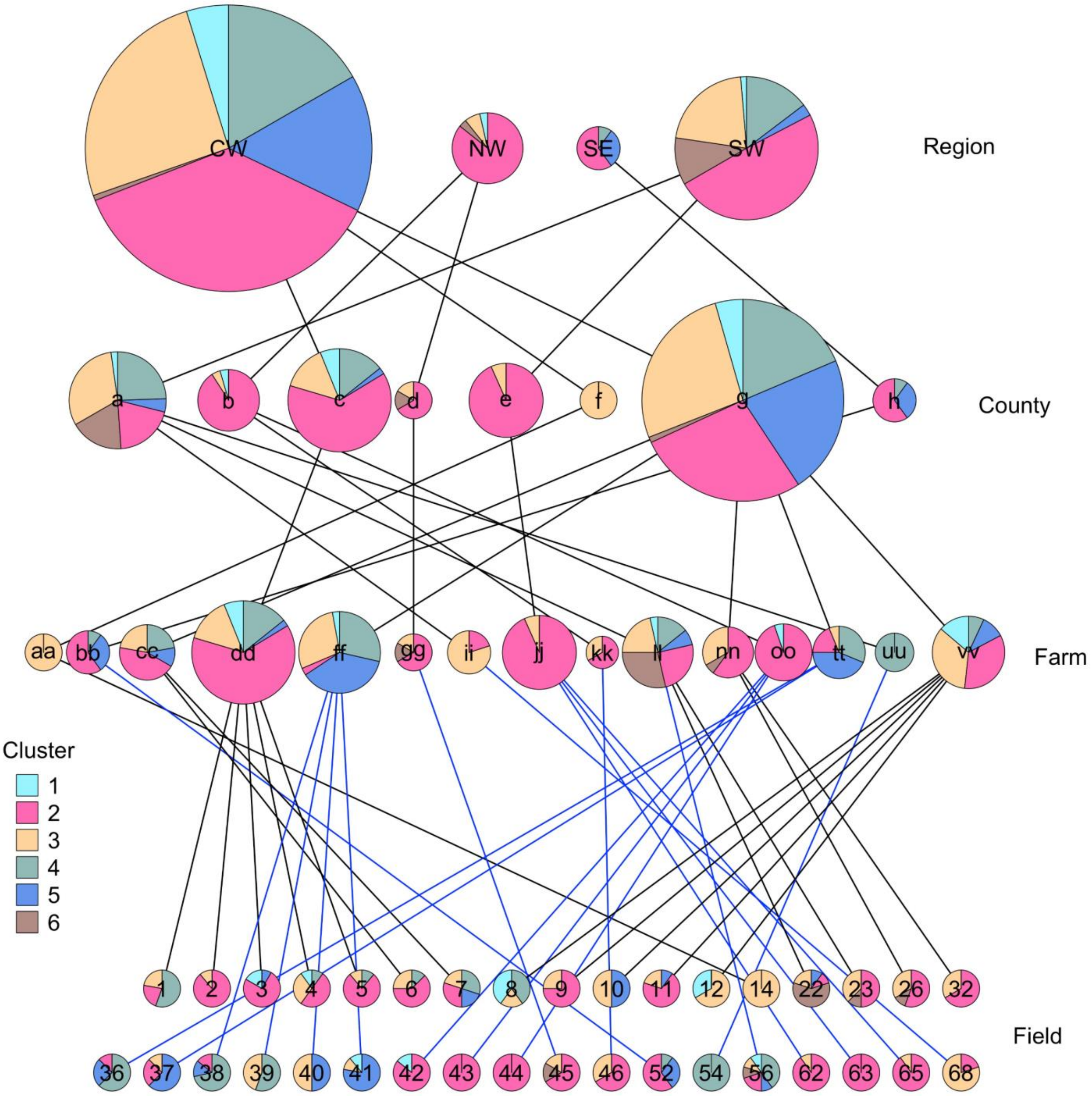

Supplementary figure 2. Network showing the distribution of core gene clusters across the tomato production system for variables region, county, farm, and field. Nodes (in rows) represent categories for each variable and links indicate hierarchical associations. All 281 strains from the collection are represented for each variable. Node size is proportional to the number of strains evaluated for a category, and the pie chart indicates the proportion of each core gene cluster. Black versus blue links from farms to fields distinguish fields that are in the top and bottom row, respectively.

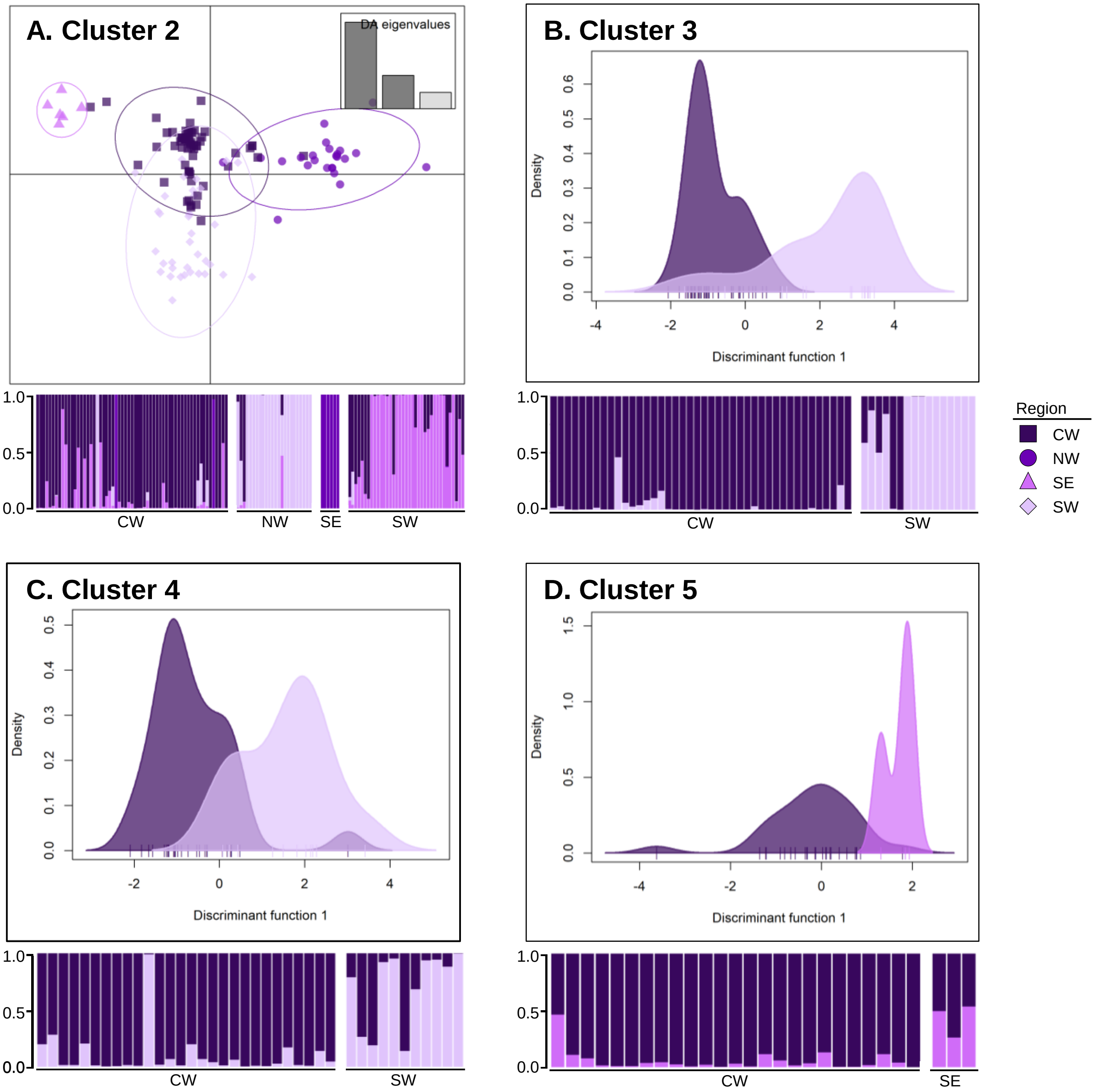

E

Cluster	AMOVA	P value
2	9.59%	0.002
3	9.43%	0.001
4	2.50%	0.001
5	6.39%	0.008

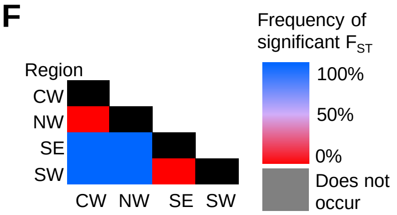

Supplementary figure 3. Population differentiation of *Xanthomonas perforans* across associated regions. Differentiation was based on chromosomal SNPs compared to the respective reference genome (Xp2010 for cluster 2, Xp17-12 for cluster 3, and 91-118 for clusters 4 and 5). Subfigures A-D depict subdivision of all regions according to discriminant analyses of principal components (DAPC) and associated population membership probabilities across clusters 2-5, with 26, 12, 6, and 2 principal components, respectively. Points and bars on DAPC plots and corresponding population membership probability plots, respectively, represent individual strains. DAPC plot points are surrounded by 95% inertia ellipses, and colors and shapes denote region association. Analysis of molecular variants (AMOVA) was calculated for samples within each DAPC plot (E). Variance in allele frequencies among populations (i.e. F_{ST}) was calculated using the Tamura distance model. Matrix colors depict the frequency of a significant F_{ST} value (p value = 0.05) across all specific region pairwise occurrences for core gene clusters 2, 3, 4, and 5 (F).

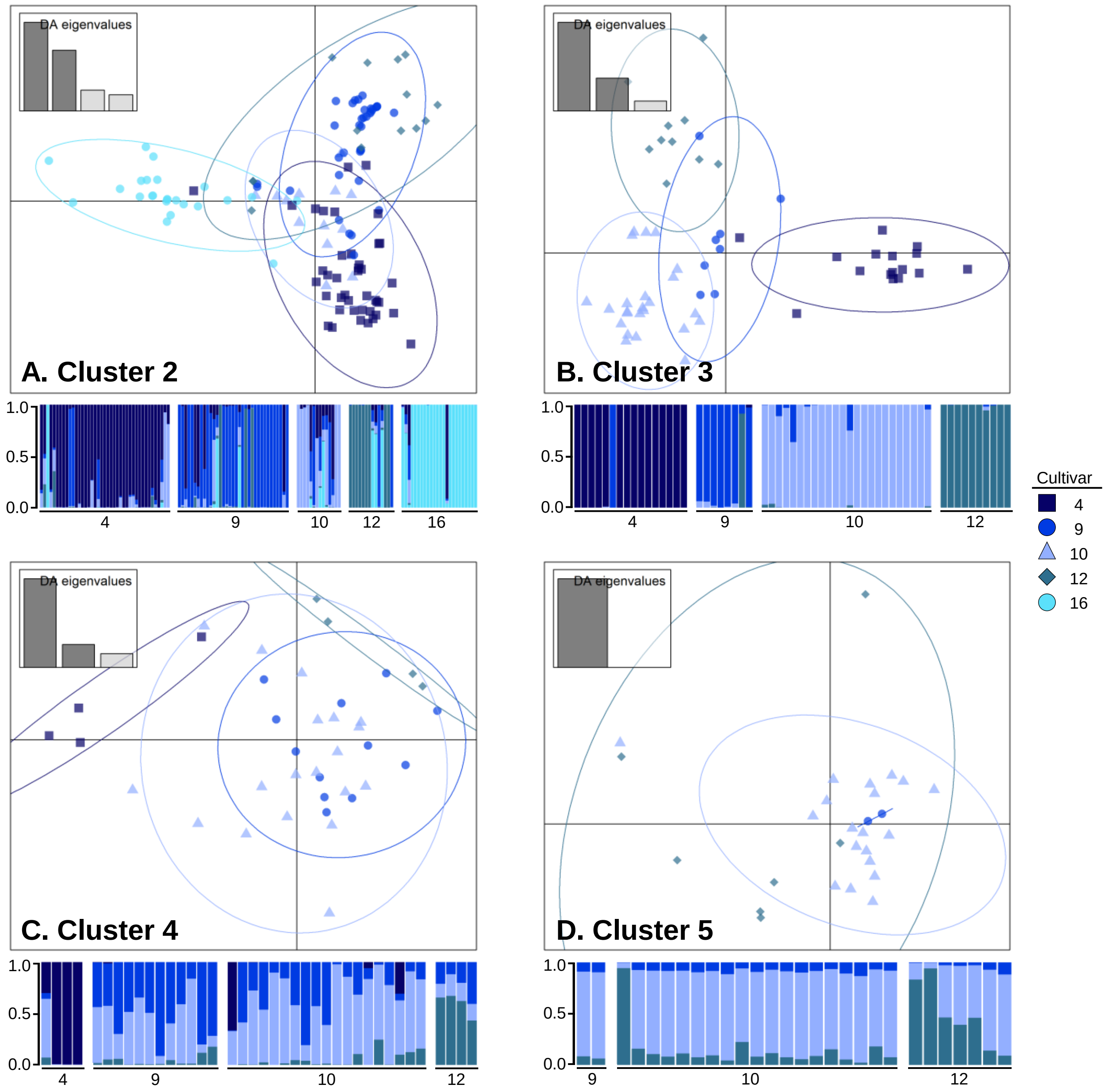

E

Cluster	AMOVA	P value
2	9.04%	0.001
3	10.18%	0.001
4	2.89%	0.001
5	2.17%	0.047

F

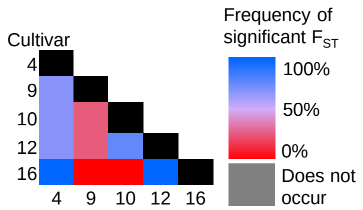

Supplementary figure 4. Population differentiation of *Xanthomonas perforans* across associated cultivars. Differentiation was based on chromosomal SNPs compared to the respective reference genome (Xp2010 for cluster 2, Xp17-12 for cluster 3, and 91-118 for clusters 4 and 5). Subfigures A-D depict subdivision of all cultivars according to discriminant analyses of principal components (DAPC) and associated population membership probabilities across clusters 2-5, with 26, 12, 6, and 2 principal components, respectively. Points and bars on DAPC plots and corresponding population membership probability plots, respectively, represent individual strains. DAPC plot points are surrounded by 95% inertia ellipses, and colors and shapes denote cultivar association. Analysis of molecular variants (AMOVA) was calculated for samples within each DAPC plot (E). Variance in allele frequencies among populations (i.e. F_{ST}) was calculated using the Tamura distance model. Matrix colors depict the frequency of a significant F_{ST} value (p value = 0.05) across all specific cultivar pairwise occurrences for core gene clusters 2, 3, 4, and 5 (F).

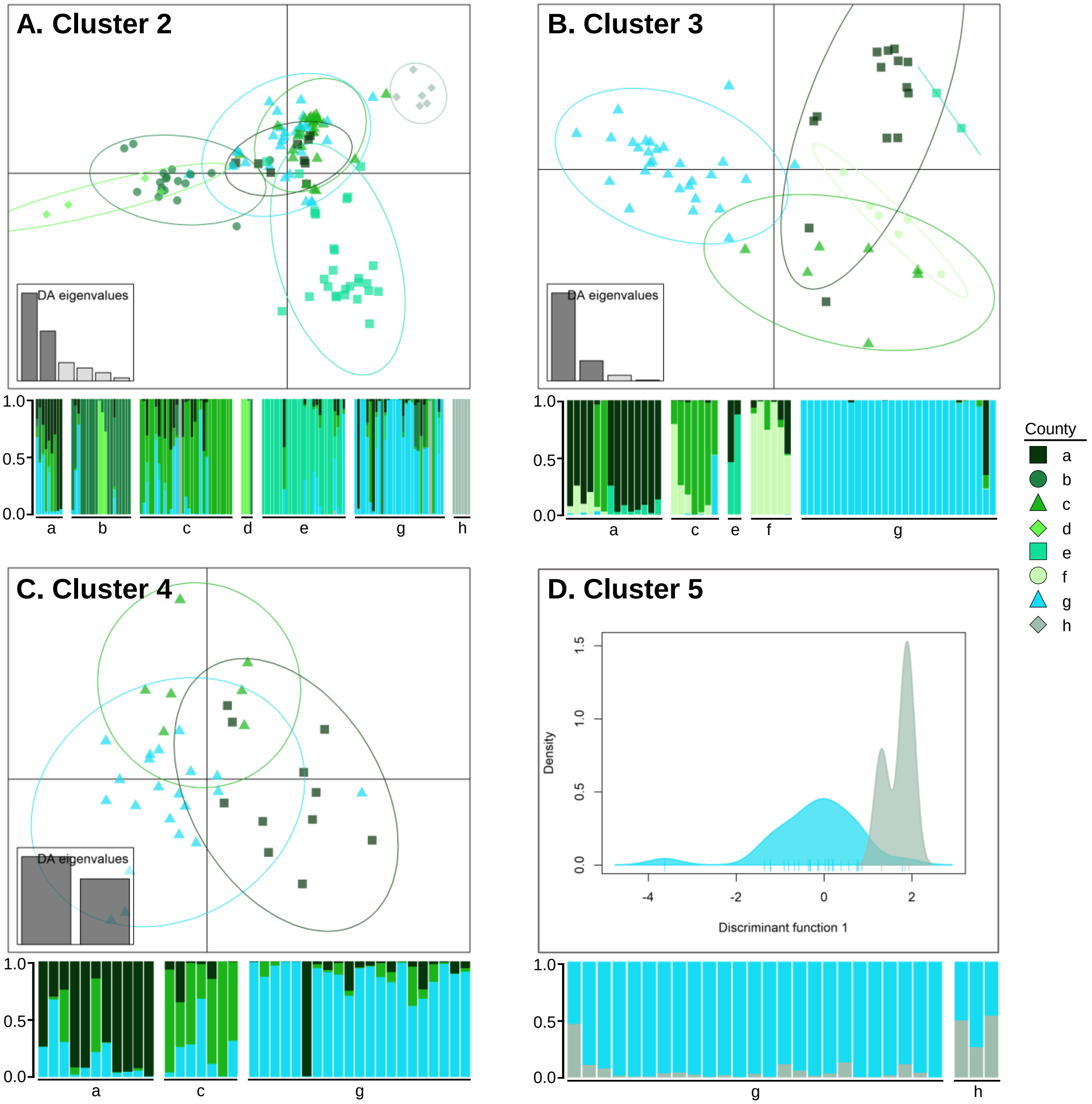

E

Cluster	AMOVA	P value
2	12.53%	0.001
3	9.62%	0.010
4	2.75%	0.001
5	6.39%	0.008

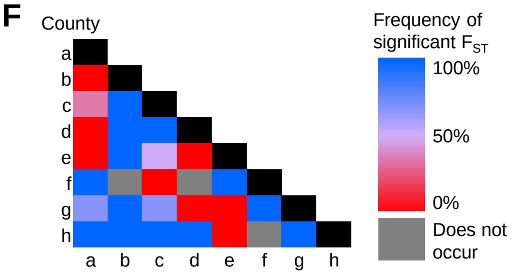

Supplementary figure 5. Population differentiation of *Xanthomonas perforans* across associated counties. Differentiation was based on chromosomal SNPs compared to the respective reference genome (Xp2010 for cluster 2, Xp17-12 for cluster 3, and 91-118 for clusters 4 and 5). Subfigures A-D depict subdivision of all counties according to discriminant analyses of principal components (DAPC) and associated population membership probabilities across clusters 2-5, with 26, 12, 6, and 2 principal components, respectively. Points and bars on DAPC plots and corresponding population membership probability plots, respectively, represent individual strains. DAPC plot points are surrounded by 95% inertia ellipses, and colors and shapes denote county association. Analysis of molecular variants (AMOVA) was calculated for samples within each DAPC plot (E). Variance in allele frequencies among populations (i.e. F_{ST}) was calculated using the Tamura distance model. Matrix colors depict the frequency of a significant F_{ST} value (p value = 0.05) across all specific county pairwise occurrences for core gene clusters 2, 3, 4, and 5 (F).

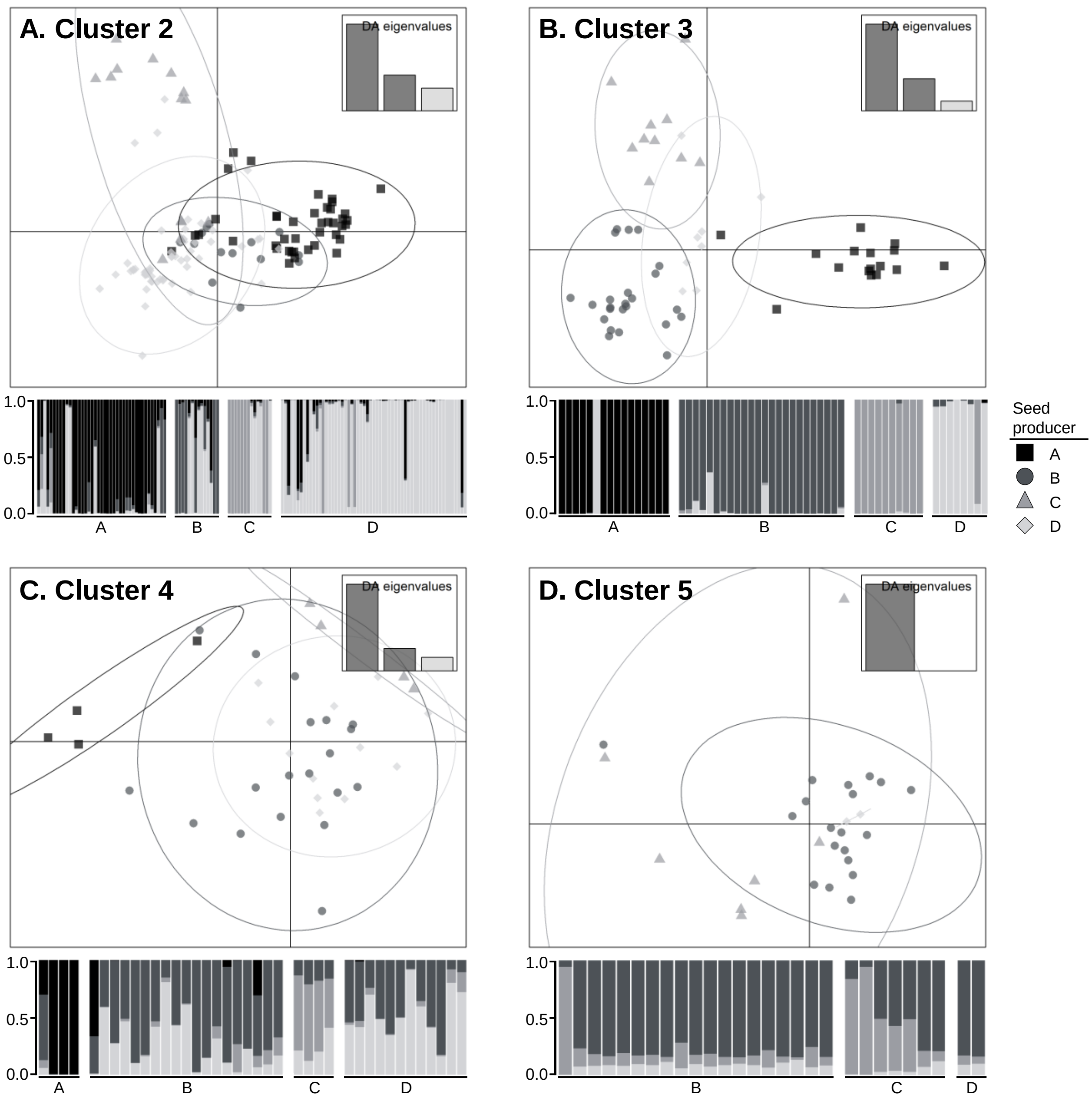

E

Cluster	AMOVA	P value
2	10.20%	0.001
3	10.18%	0.001
4	2.89%	0.001
5	2.17%	0.047

F

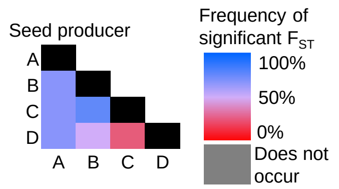

Supplementary figure 6. Population differentiation of *Xanthomonas perforans* across associated seed producers. Differentiation was based on chromosomal SNPs compared to the respective reference genome (Xp2010 for cluster 2, Xp17-12 for cluster 3, and 91-118 for clusters 4 and 5). Subfigures A-D depict subdivision of all seed producers according to discriminant analyses of principal components (DAPC) and associated population membership probabilities across clusters 2-5, with 26, 12, 6, and 2 principal components, respectively. Points and bars on DAPC plots and corresponding population membership probability plots, respectively, represent individual strains. DAPC plot points are surrounded by 95% inertia ellipses, and colors and shapes denote seed producer association. Analysis of molecular variants (AMOVA) was calculated for samples within each DAPC plot (E). Variance in allele frequencies among populations (i.e. F_{ST}) was calculated using the Tamura distance model. Matrix colors depict the frequency of a significant F_{ST} value (p value = 0.05) across all specific seed producer pairwise occurrences for core gene clusters 2, 3, 4, and 5 (F).

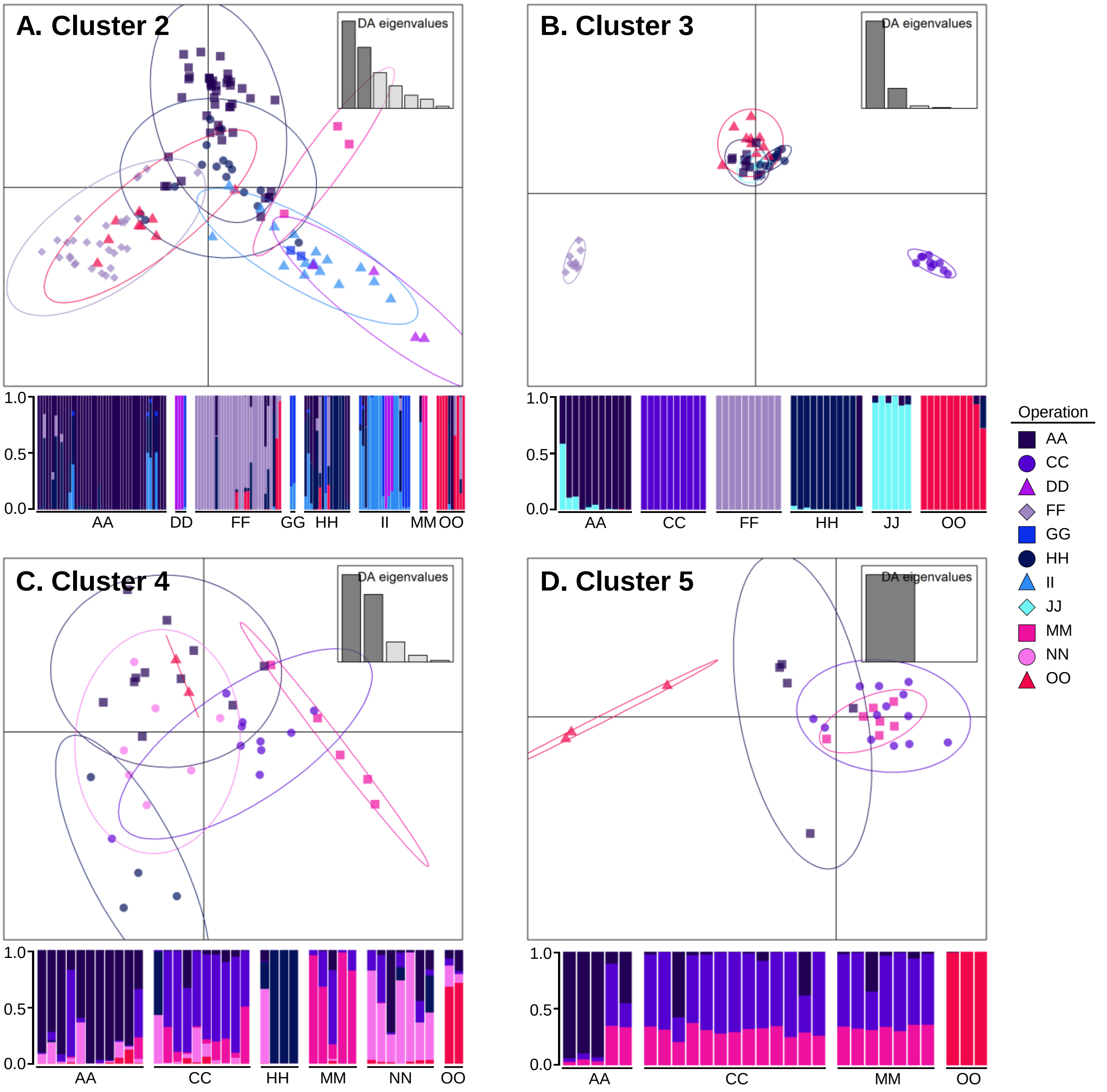

E

Cluster	AMOVA	P value
2	13.18%	0.002
3	20.73%	0.001
4	4.80%	0.001
5	4.31%	0.002

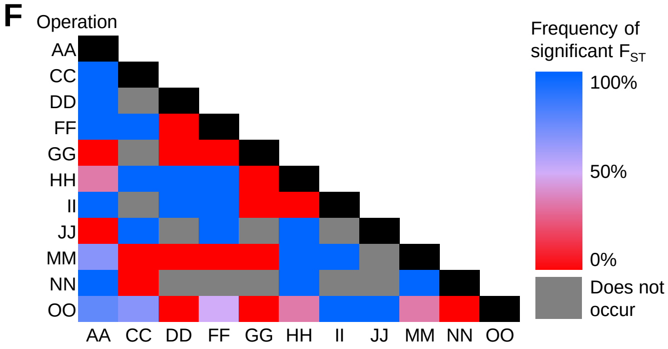

Supplementary figure 7. Population differentiation of *Xanthomonas perforans* across associated grower operations. Differentiation was based on chromosomal SNPs compared to the respective reference genome (Xp2010 for cluster 2, Xp17-12 for cluster 3, and 91-118 for clusters 4 and 5). Subfigures A-D depict subdivision of all grower operations according to discriminant analyses of principal components (DAPC) and associated population membership probabilities across clusters 2-5, with 26, 12, 6, and 2 principal components, respectively. Points and bars on DAPC plots and corresponding population membership probability plots, respectively, represent individual strains. DAPC plot points are surrounded by 95% inertia ellipses, and colors and shapes denote grower operation association. Analysis of molecular variants (AMOVA) was calculated for samples within each DAPC plot (E). Variance in allele frequencies among populations (i.e. F_{ST}) was calculated using the Tamura distance model. Matrix colors depict the frequency of a significant F_{ST} value (p value = 0.05) across all specific grower operation pairwise occurrences for clusters 2, 3, 4, and 5 (F).

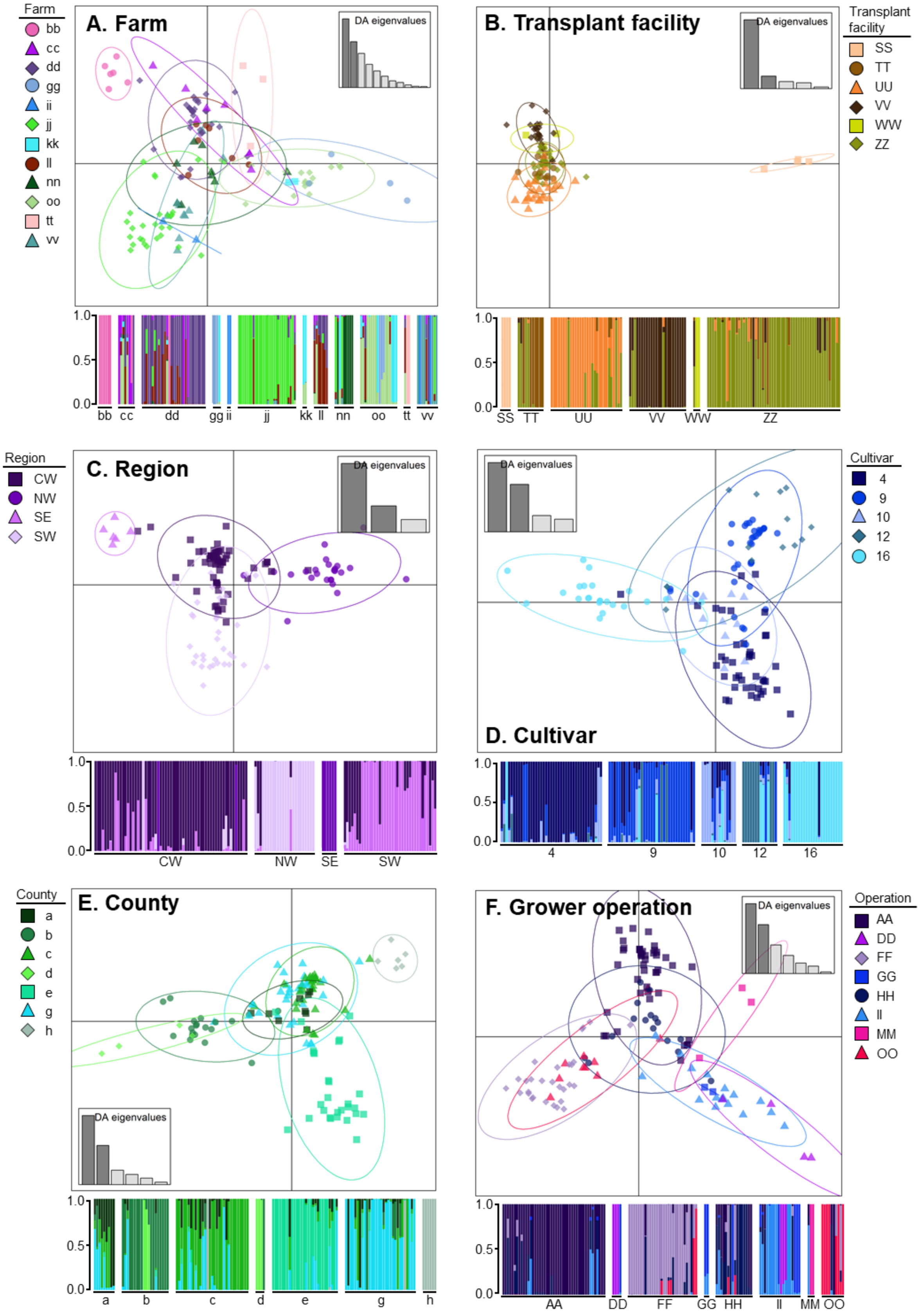

Supplementary figure 8. Population differentiation of *Xanthomonas perforans* gene cluster 2 strains across associated tomato production system variables. Differentiation was based on chromosomal SNPs compared to the reference genome Xp2010. Subfigures depict subdivision of the variables farm (A), transplant facility (B), region (C), cultivar (D), county (E), and grower operation (F) according to discriminant analyses of principal components (DAPC) and associated population membership probabilities with 26 principal components. Points and bars on DAPC plots and corresponding population membership probability plots, respectively, represent individual strains. DAPC plot points are surrounded by 95% inertia ellipses, and colors and shapes denote category association.

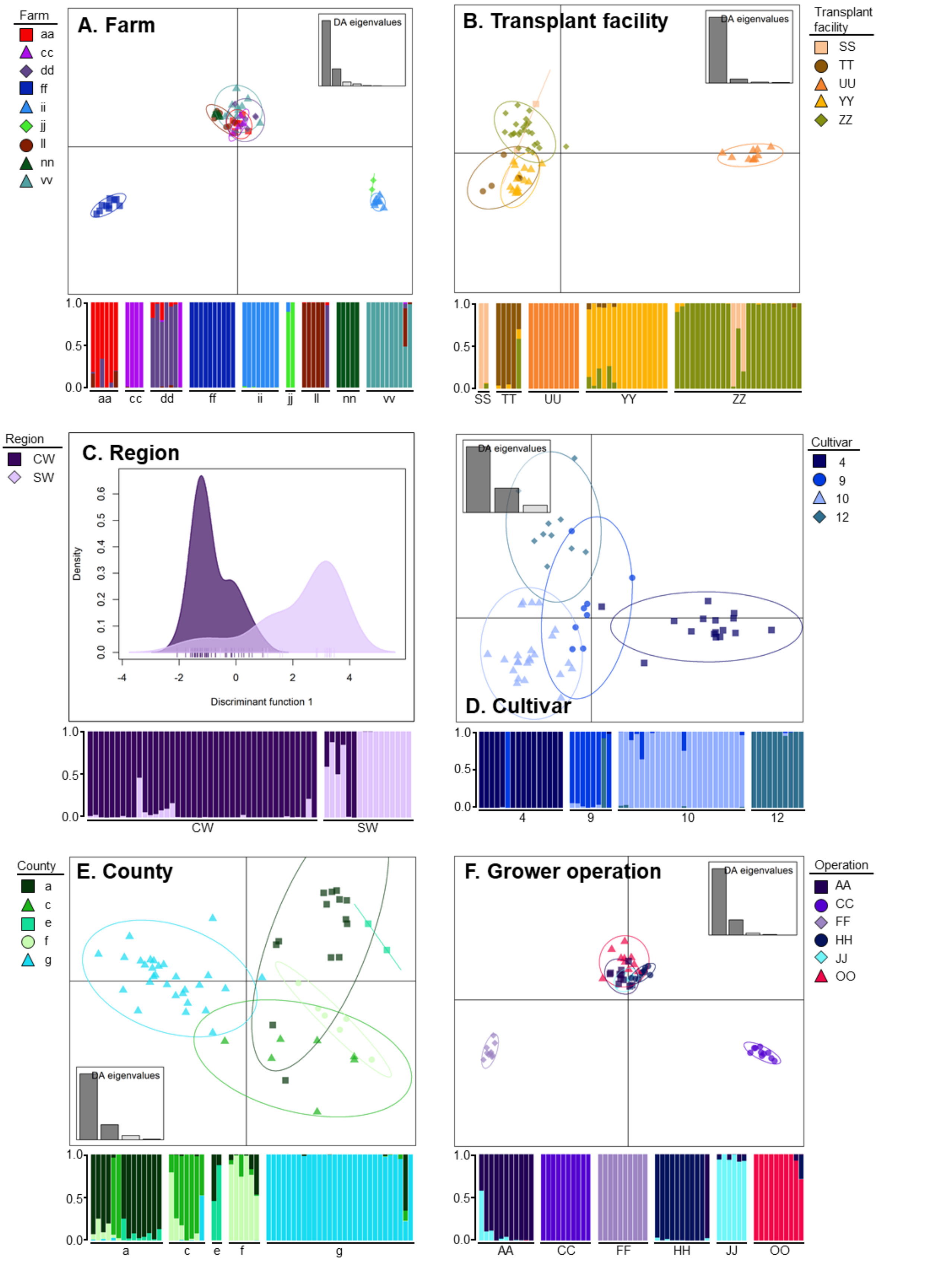

Supplementary figure 9. Population differentiation of *Xanthomonas perforans* gene cluster 3 strains across associated tomato production system variables. Differentiation was based on chromosomal SNPs compared to the reference genome Xp17-12. Subfigures depict subdivision of the variables farm (A), transplant facility (B), region (C), cultivar (D), county (E), and grower operation (F) according to discriminant analyses of principal components (DAPC) and associated population membership probabilities with 12 principal components. Points and bars on DAPC plots and corresponding population membership probability plots, respectively, represent individual strains. DAPC plot points are surrounded by 95% inertia ellipses, and colors and shapes denote category association.

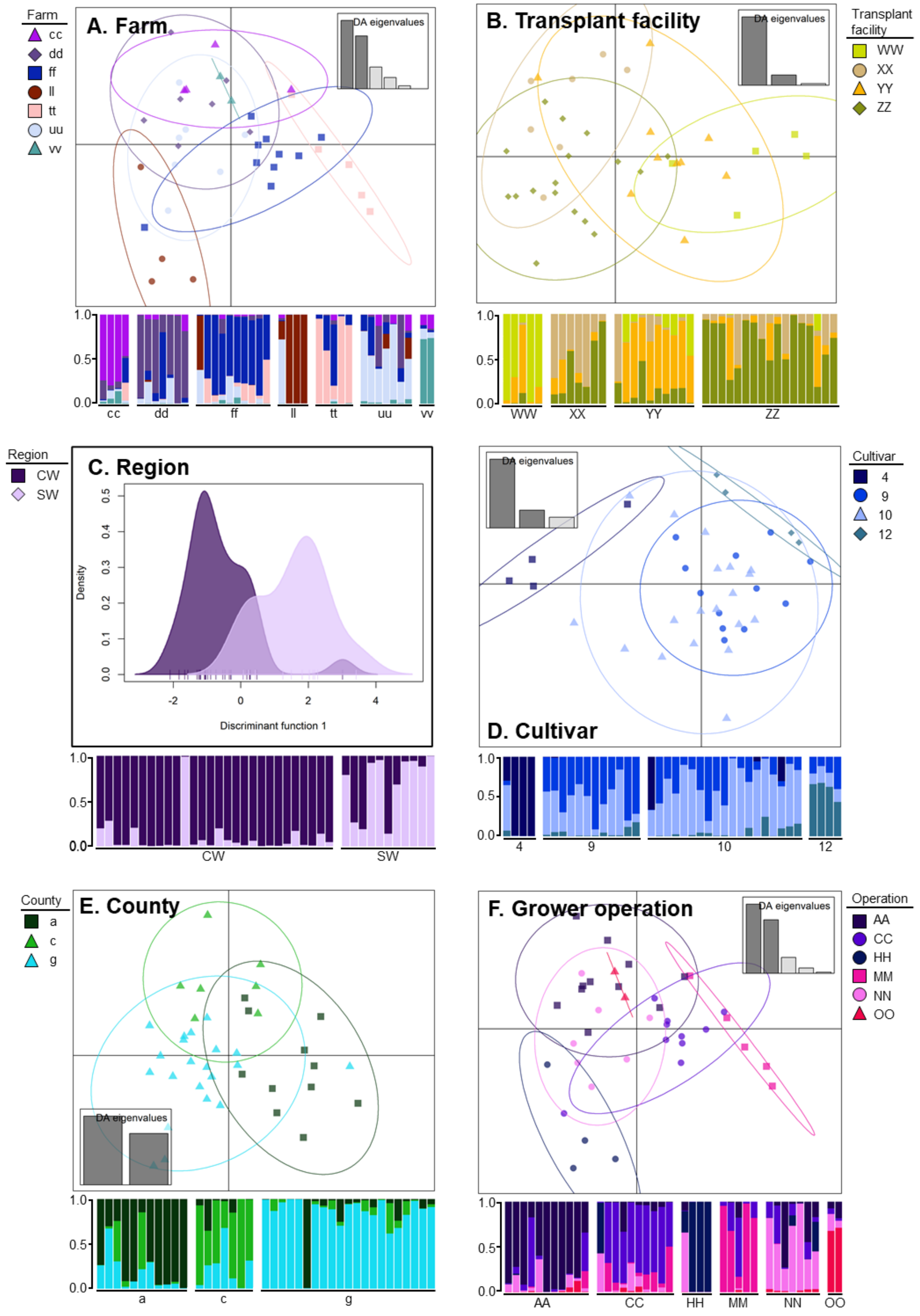

Supplementary figure 10. Population differentiation of *Xanthomonas perforans* gene cluster 4 strains across associated tomato production system variables. Differentiation was based on chromosomal SNPs compared to the reference genome 91-118. Subfigures depict subdivision of the variables farm (A), transplant facility (B), region (C), cultivar (D), county (E), and grower operation (F) according to discriminant analyses of principal components (DAPC) and associated population membership probabilities with 6 principal components. Points and bars on DAPC plots and corresponding population membership probability plots, respectively, represent individual strains. DAPC plot points are surrounded by 95% inertia ellipses, and colors and shapes denote category association.

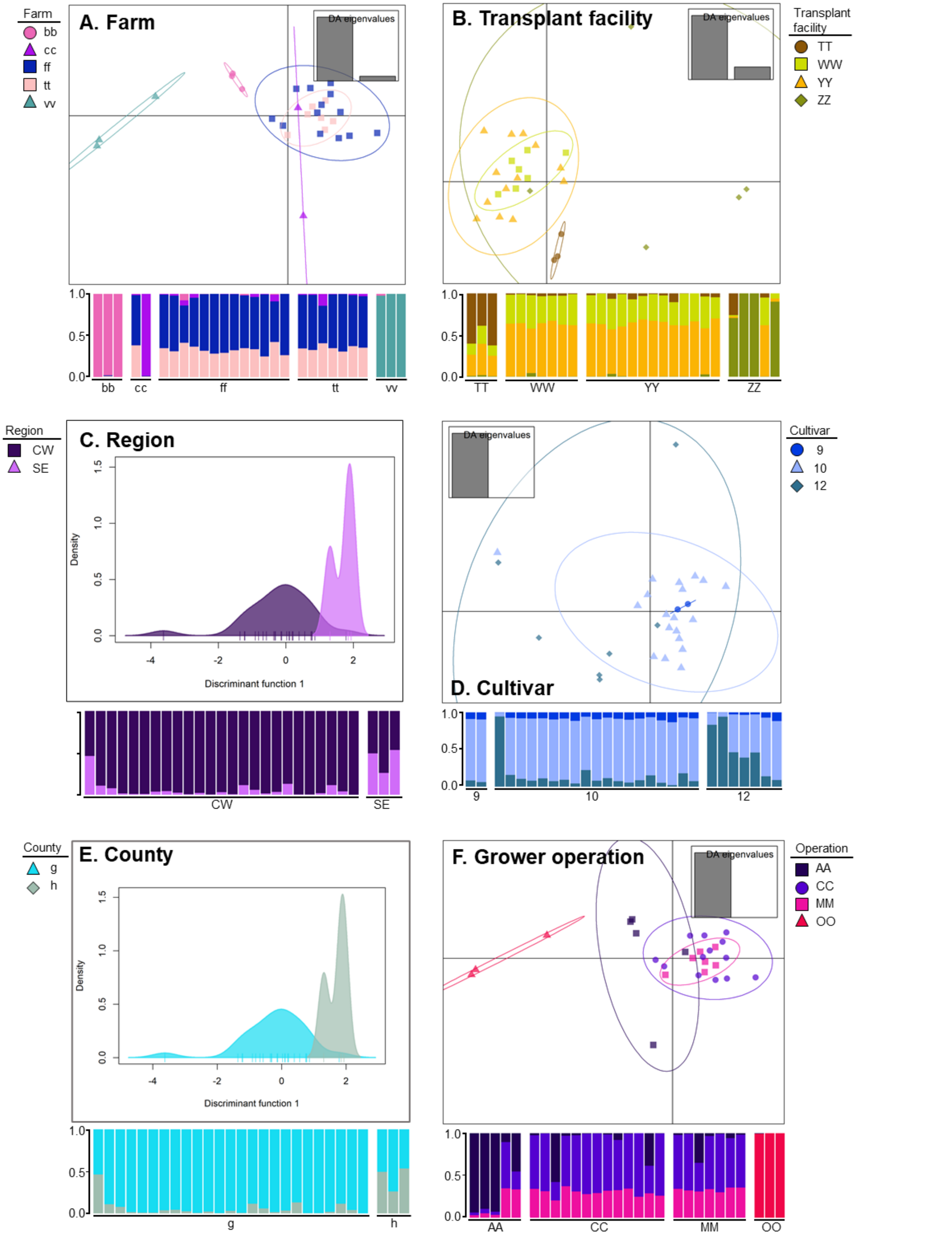

Supplementary figure 11. Population differentiation of *Xanthomonas perforans* gene cluster 5 strains across associated tomato production system variables. Differentiation was based on chromosomal SNPs compared to the reference genome 91-118. Subfigures depict subdivision of the variables farm (A), transplant facility (B), region (C), cultivar (D), county (E), and grower operation (F) according to discriminant analyses of principal components (DAPC) and associated population membership probabilities with 2 principal components. Points and bars on DAPC plots and corresponding population membership probability plots, respectively, represent individual strains. DAPC plot points are surrounded by 95% inertia ellipses, and colors and shapes denote category association.
